# ^13^C MRS and LC–MS Flux Analysis of Tumor Intermediary Metabolism

**DOI:** 10.3389/fonc.2016.00135

**Published:** 2016-06-15

**Authors:** Alexander A. Shestov, Seung-Cheol Lee, Kavindra Nath, Lili Guo, David S. Nelson, Jeffrey C. Roman, Dennis B. Leeper, Mariusz A. Wasik, Ian A. Blair, Jerry D. Glickson

**Affiliations:** ^1^Laboratory of Molecular Imaging, Department of Radiology, Perelman School of Medicine, University of Pennsylvania, Philadelphia, PA, USA; ^2^Department of Systems Pharmacology and Translational Therapeutics, Center for Cancer Pharmacology, Perelman School of Medicine, University of Pennsylvania, Philadelphia, PA, USA; ^3^Department of Radiation Oncology, Thomas Jefferson University, Philadelphia, PA, USA; ^4^Laboratory Medicine, Department of Pathology, Perelman School of Medicine, University of Pennsylvania, Philadelphia, PA, USA; ^5^Department of Biochemistry and Biophysics, Perelman School of Medicine, University of Pennsylvania, Philadelphia, PA, USA

**Keywords:** metabolic modeling, sensitivity analysis, cancer metabolism, isotopomer, metabolic flux analysis, isotopolome, ^13^C magnetic resonance spectroscopy, ^13^C liquid chromatography-mass spectrometry

## Abstract

We present the first validated metabolic network model for analysis of flux through key pathways of tumor intermediary metabolism, including glycolysis, the oxidative and non-oxidative arms of the pentose pyrophosphate shunt, the TCA cycle as well as its anaplerotic pathways, pyruvate–malate shuttling, glutaminolysis, and fatty acid biosynthesis and oxidation. The model that is called Bonded Cumomer Analysis for application to ^13^C magnetic resonance spectroscopy (^13^C MRS) data and Fragmented Cumomer Analysis for mass spectrometric data is a refined and efficient form of isotopomer analysis that can readily be expanded to incorporate glycogen, phospholipid, and other pathways thereby encompassing all the key pathways of tumor intermediary metabolism. Validation was achieved by demonstrating agreement of experimental measurements of the metabolic rates of oxygen consumption, glucose consumption, lactate production, and glutamate pool size with independent measurements of these parameters in cultured human DB-1 melanoma cells. These cumomer models have been applied to studies of DB-1 melanoma and DLCL2 human diffuse large B-cell lymphoma cells in culture and as xenografts in nude mice at 9.4 T. The latter studies demonstrate the potential translation of these methods to *in situ* studies of human tumor metabolism by MRS with stable ^13^C isotopically labeled substrates on instruments operating at high magnetic fields (≥7 T). The melanoma studies indicate that this tumor line obtains 51% of its ATP by mitochondrial metabolism and 49% by glycolytic metabolism under both euglycemic (5 mM glucose) and hyperglycemic conditions (26 mM glucose). While a high level of glutamine uptake is detected corresponding to ~50% of TCA cycle flux under hyperglycemic conditions, and ~100% of TCA cycle flux under euglycemic conditions, glutaminolysis flux and its contributions to ATP synthesis were very small. Studies of human lymphoma cells demonstrated that inhibition of mammalian target of rapamycin (mTOR) signaling produced changes in flux through the glycolytic, pentose shunt, and TCA cycle pathways that were evident within 8 h of treatment and increased at 24 and 48 h. Lactate was demonstrated to be a suitable biomarker of mTOR inhibition that could readily be monitored by ^1^H MRS and perhaps also by FDG-PET and hyperpolarized ^13^C MRS methods.

## Introduction

Tumor intermediary metabolism provides the energy required to sustain cancer cells over their entire life span. This energy is utilized to maintain cation pumps, including the pumps that eliminate cytotoxic drugs contributing to multi-drug resistance. It also provides energy for cellular motility during metastasis. Tumor metabolism also provides key metabolic precursors for DNA, protein, and lipid synthesis during cellular replication. Delineation of flux through these critical metabolic pathways provides an invaluable tool, complementary to genomic information, for cancer diagnosis as well as prediction and early detection of therapeutic response. Tracing these metabolic pathways was first accomplished through the use of radioactive isotopic labels, but monitoring of flux through these pathways has more recently been achieved by analysis of stable ^13^C isotope kinetics labeling detected with metabolomic tools – magnetic resonance spectroscopy (MRS) and liquid chromatography–mass spectrometry (LC–MS) – the former providing a potentially non-invasive but relatively insensitive method for imaging metabolism and the latter providing a much more sensitive but invasive approach. Analysis of these dynamic isotopolome (labeled metabolome) data used to require solution of hundreds of differential equations that could only be accomplished with the help of supercomputers (1), but today can be readily accomplished with laptop computers virtually in real time. This problem has been solved for normal organs, such as the heart (1–6), brain (7–9), and liver (10–12); our goal here is to describe the first validated solution of the problem for tumors.

We present data on human DB-1 melanoma and models of various non-Hodgkin’s lymphomas (NHLs). DB-1 melanoma has served as the principal tumor model on which this technology has been developed and validated (13, 14). This technology has been applied to isolated perfused tumor cells grown *ex vivo* as monolayers on solid microcarrier beads in a bioreactor system or in flasks and to *in vivo* xenografts in mice. Isotope exchange has been monitored non-invasively *in vivo* or in intact cells by ^13^C MRS and invasively by LC–MS following *ex vivo* extraction. The NHL models have been examined by similar methods except that because they are anchorage independent, they had to be immobilized by encapsulation in agarose beads or studied in batch suspension culture. Encapsulation of cells in agarose or alginate beads invariably introduces heterogeneity in the cellular microenvironment, a problem that can be overcome by growth in batch culture from which aliquots are isolated for periodic analysis. The goal is to lay the groundwork for performing these measurements non-invasively on human patients who will be monitored in high-field (≥7 T) spectrometers before and following treatment with appropriate therapeutic agents whose choice will be at least partially dictated by metabolic flux analysis of tumors following administration of appropriate ^13^C-labeled substrates. Changes in tumor metabolism indicated by these labeling experiments will point to the probable success or failure of drug delivery and will monitor the effect of these agents on tumor metabolism.

Non-Hodgkin’s lymphoma tumors have served as models for developing methods for monitoring response to signal-transduction pathway inhibitors (15) as well as response to more conventional drug combinations, such as RCHOP [Rituximab, Cyclophosphamide, doxorubicin hydrochloride (Hydroxydaunomycin), vincristine sulfate (Oncovin), and Prednisone (16, 17)]. In this review, we will focus on studies of mammalian target of rapamycin (mTOR) inhibitors as representative of the former class of targeted therapeutic agents. Drugs that are inhibitors of signal-transduction pathways are generally more cytostatic rather than cytotoxic and usually do not exhibit significant changes in tumor volume except very late in the course of therapy. For these drugs, monitoring their effect on tumor metabolism may prove to be the most efficient approach to detect therapeutic response. Since ^13^C metabolomics/isotopolomic studies are expensive and labor intensive, it may prove advantageous to identify surrogate biomarkers of therapeutic response whose metabolism could be followed by more conventional methods, such as lactate imaging by ^1^H MRS or ^1^H chemical exchange of saturation-transfer (^1^H CEST), hyperpolarized ^13^C MRS or PET/CT with FDG or other appropriate metabolic probes. This two-stage approach – utilizing ^13^C MRS for initial exploration of overall tumor metabolism and identification of suitable biomarkers for subsequent follow-up by ^1^H MRS or other appropriate method – is a general strategy that we are proposing for monitoring signal-transduction inhibitors. The feasibility of achieving this “personalized medicine dream” or at least the ^13^C MRS exploratory phase is already demonstrated through the pioneer work of investigators at the University of Texas Southwestern, who have monitored the metabolism of ^13^C-labeled substrates by *ex vivo* analysis of surgical specimens obtained from patients with brain (18) and lung (19) tumors. Studies of human tumor xenografts in mice detected by ^13^C MRS (see below) will further demonstrate the potential feasibility of non-invasive monitoring of tumor metabolism. The purpose of this manuscript is to describe the technical details on how ^13^C isotope kinetics can be monitored and quantitatively analyzed.

## Background

### Types of ^13^C MRS Experiments

Following administration of ^13^C-labeled substrates, one can monitor the time course of distribution of the isotopic label among the various metabolites of the substrate either directly by ^13^C MRS or indirectly through ^1^H MRS of the protons directly bonded to the isotopically labeled carbon atoms. These methods have been extensively reviewed by de Graaf et al. (20) and will not be considered further here except to note their key advantages and limitations. Direct detection offers the advantage of simultaneously monitoring all the sites of isotopic substitution, but signal intensity will depend on the detection coil, the method used for proton decoupling as well as on the pulse sequence that is used for ^13^C excitation. Broadband decoupling, the method that we have employed, will simultaneously decouple all the protons and will produce nuclear Overhauser enhancements (NOE) that will depend on relaxation times, the fraction of the relaxation occurring through dipolar mechanisms and the broadband decoupling technique (e.g., MLEV and WALTZ). There will be differences in signal enhancement, and these will have to be calibrated. For *in vivo* applications, the NOEs vary between 1.3 and 2.9 for protonated carbons, and are 1.0 for unprotonated carbons or carbons that relax by non-dipolar mechanisms. Alternatively, one could use dynamic polarization-transfer excitation for direct ^13^C excitation (21, 22); this method requires quantum mechanical density matrix or product-operator analysis for quantitative interpretation, which is beyond the scope of this manuscript. In general, the direct excitation methods, delineated as ^13^C-{^1^H}, are less sensitive than the indirect detection techniques, ^1^H-{^13^C}, by which the protons directly bonded to the isotopically labeled carbon atoms are monitored with the ^13^C magnetization being manipulated either by simple subtraction of multiple experiments, by multiple quantum coherence transfer or by J-difference pulse sequences that depend on spin–spin coupling (20). Another key consideration in these experiments is the issue of power deposition or sample heating, which becomes limiting in the inverse-detection techniques when broadband decoupling of carbon resonances is applied and also depends on coil design and various other factors. While we note the potential signal enhancing possibilities that can be achieved with indirect detection, most investigators, and our group included, utilize simple direct ^13^C-{^1^H} detection with broadband decoupling at least as a starting point for MRS studies of ^13^C isotope exchange kinetics.

### Metabolic Flux-Analysis Models

Metabolic flux-analysis dates back to the classic study published in 1983 by Chance, Seeholtzer, Kobayashi, and Williamson at the University of Pennsylvania and University College London, on flux through the tricarboxylic acid cycle (TCA cycle) in Langendorff perfused rat hearts (1). These investigators sacrificed large numbers of rats, excised their hearts, mounted them in a Langendorff retrograde perfusion apparatus, perfused the hearts with various ^13^C enriched substrates ([3-^13^C] pyruvate or [2-^13^C] acetate), freeze clamped the hearts at various time points, extracted the water soluble metabolites with perchloric acid, and analyzed the extracts with direct ^13^C-{1H} MRS to measure the time course of isotopic labeling of glutamate, alanine, and aspartate. The data were fit by least-squares analysis to an idealized TCA cycle model consisting of oxaloacetate in equilibrium with aspartate, citrate, α-ketoglutarate, and reactions involving glutamate, succinate, and malate. The analysis required about 325 differential equations that were solved with the aid of a Cray supercomputer. The model fit estimates of isotope enrichment of carbon atoms in glutamate and aspartate, and calculated the rate of TCA cycling, but did not attempt to validate this rate. This was accomplished later by Chatham et al. (2), who calculated the metabolic rate of oxygen consumption (MRO_2_) from the rate of production of reducing equivalents assuming that the system was at equilibrium. They compared the calculated rate with the experimental rate measured with oxygen electrodes placed at the inlet and outlet ports of the Langendorff perfusion apparatus; MRS and MRO_2_ data acquisition were performed directly in the magnet on the beating heart, and the mathematical fitting was performed with a simple laptop computer. The results demonstrated that the original model proposed by Chance et al. (1) consistently predicted MRO_2_ values that were significantly below the experimentally measured rates. The error was attributed to the absence of the shuttles in the TCA cycle model; inclusion of the malate-aspartate and glycerophosphate shuttles in the model led to perfect agreement between experimental and model-predicted data. We believe that this was the first validated isotope enrichment model.

In 1994, Sherry and Malloy and coworkers at Dallas Southwestern (23) introduced a considerably more efficient method, isotopomer analysis, which took into account multiple sites of isotope labeling and substantially simplified the mathematics of analysis of the kinetics of isotope labeling. This method was first employed to measure relative rates of anaplerotic vs. TCA cycle flux in the heart and was later extended to monitor more extensive metabolic pathways in various organs, including liver (24–30) and brain (31–42). Comparison of experimental and predicted MRO_2_ values validated the cardiac isotopomer analysis model of Jeffrey et al. (3).

The motivation for developing additional theoretical framework to study the fluxome (13) came from the challenges of modern metabolic flux analysis. The details of flux analysis based on cumomers were introduced in Ref. (43). Alternative cumomer methods, such as elementary metabolite units (44), aim to minimize the dimension of variables space and to reduce the cost of computation.

More recently, Shestov and colleagues introduced bonded cumomer analysis at the University of Minnesota, where research focused primarily on the brain (45), and later at the University of Pennsylvania, where tumors were the central focus of research (14, 46). Cumomer analysis is a more efficient method of generating the various isotopomer equations. Two variants of the cumomer method are still under development, bonded cumomer analysis, which applies to the analysis of ^13^C MRS data (14, 46), and fragmented cumomer analysis (47–49) that analyzes LC–MS data. The relative efficiency of the cumomer methods is perhaps best illustrated by the fact that the original study of TCA cycle metabolism of the heart using a model that lacked inclusion of the two shuttles required about 325 differential equations to solve, whereas the current bonded cumomer model describing about 10 pathways of tumor intermediary metabolism including the TCA cycle and the pentose phosphate pathway are accomplished with ~210 differential equations that can be solved virtually in real time with an ordinary laptop computer.

### Types of ^13^C Metabolic Models

Metabolic isotope kinetic models can be subdivided into steady-state and dynamic models, with the former analyzing measurements of isotope distribution after isotopic steady state has been established and the latter requiring a complete kinetic study. The kinetic analysis is much more demanding and is yet to be achieved in the clinical arena (18, 19). Steady-state measurements can only measure relative rates, normalized to some standard reference rate, such as TCA cycle flux, whereas the true kinetic measurements yield absolute fluxes through individual metabolic pathways. Steady-state measurements can often be made on surgically excised or biopsy derived specimens, and in the case of MRS measurements, their signal-to-noise ratio can be enhanced by long-term signal averaging, but kinetic measurements have to be performed non-invasively in real time by MRS studies of isolated perfused cells grown in a suitable bioreactor contained in the NMR spectrometer or by non-invasive MRS measurements on living animals or humans. Alternatively, one can perform kinetic experiments by periodically sampling cells or extracts from a living host or from a bioreactor system. Both types of measurements may eventually prove useful in the clinic, but the hope is that high-field MRS will for the first time enable totally non-invasive studies of human subjects. Dynamic nuclear polarization already enables performance of true kinetic measurements in living animals and even in humans, but to date this has not been accomplished at normal *in vivo* concentrations; instead, *ex vivo* injections of much higher concentrations of isotopically labeled hyperpolarized specimens, most often labeled pyruvate have been utilized and monitored within about a minute in real time. The relationship of such studies to actual *in vivo* metabolism is the subject of considerable controversy and debate that requires further investigation. Even if such methods distort *in vivo* metabolism, they might still be useful for clinical diagnosis and monitoring of disease and therapy.

### Mass Spectrometric Studies of Tumor Metabolism

For dynamic isotopic labeling, DB-1 cells were cultured in glutamine-free DMEM containing 2 mM [U-^13^C_5_,^15^N_2_] glutamine supplemented with 10% fetal bovine serum. After 0, 0.5, 1, 2, 4, and 6 h of incubation, medium was aspirated and cells were snap frozen on dry ice. For comparing euglycemic vs. hyperglycemia conditions, cells were labeled with glutamine-free DMEM containing 2 mM [U-^13^C_5_,^15^N_2_] glutamine and 5 mM glucose or 25 mM glucose for 8 h. LC–MS analysis of isotopic labeled metabolites was performed as previously described (47).

### The Metabolic Network Model

**Figure 1:**
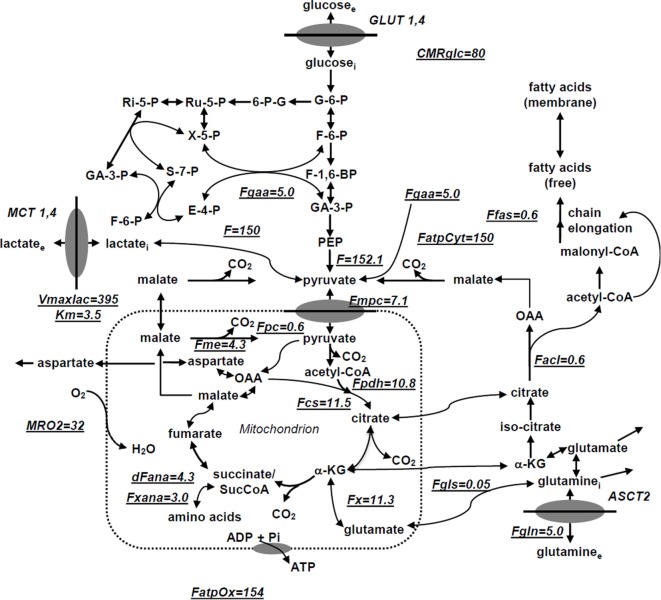
**Studied melanoma bionetwork: glycolysis, PPP, TCA cycle, ***α***-ketoglutarate-glutamate, and oxaloacetate-aspartate exchange through the malate-aspartate shuttle, anaplerosis through (a) pyruvate carboxylase activity, (b) succinyl-CoA, and (c) glutaminolysis through mitochondrial glutaminases (GLS and GLS2); pyruvate recycling through cytosolic (ME1) and mitochondrial malic enzymes (ME2), lactate dehydrogenase activity, *de novo* fatty acid synthesis, and transport processes**. Metabolic flux map and energy metabolism with predicted oxygen consumption rate MRO_2_ in the human melanoma DB1 bionetwork at hyperglycemia and normoxia. Numbers indicate fluxes in millimoles per liter-cell per hour. See Table 1 for definitions and values of the derived fluxes.

We have utilized a novel three-compartment metabolic network model with the bionetwork schematically depicted in Figure 1 to calculate fluxes through key pathways of tumor energy metabolism from ^13^C isotopic labeling data. This model includes the perfusion medium, cytosolic and mitochondrial compartments interconnected by various transporters, carriers, and enzymes that span adjacent compartments. The model is applicable to bioreactor systems and to *in vivo* data. Glucose (GLUT), monocarboxylic acid (MCT), and glutamine (GLN) transporters to the cytosolic compartment are included in the cytosolic membrane. Tumors generally express GLUT1 and GLUT3, but only GLUT1 is currently included in the model; similarly DB-1 contains MCT1 and MCT4 in approximately equal proportions (50), but since the K_M_ of MCT1 is approximately one-fifth that of MCT4 (51–53), we have only included MCT1. Because of the critical role of glutamine in tumor energetics and metabolism, we have also included the GLN transporter (ASCT2). This model can readily be extended to incorporate other isoforms of MCT and GLUT as well as transporters for other amino acids (AAs) and metabolites.

The following metabolic pathways are included in the network: glycolysis, TCA cycle, α-ketoglutarate–glutamate, and oxaloacetate–aspartate exchange through the malate-aspartate shuttle, anaplerosis through (a) pyruvate carboxylase (PC) activity, (b) at the succinyl-CoA level, and (c) glutaminolysis through mitochondrial glutaminase (GLS and GLS2), pyruvate recycling through cytosolic (ME1) and mitochondrial malic enzymes (ME2 + ME3), lactate dehydrogenase (LDH) activity, and transport processes. Oxidative and non-oxidative branches of the pentose phosphate pathway (PPP) fluxes were included in the modeling analysis as well as *de novo* lipogenesis and fatty acid (FA) oxidation.

In order to permit access of pyruvate to the pyruvate dehydrogenase complex (PDH) complex for conversion to acetyl-CoA and incorporation into the TCA cycle, we have included the mitochondrial pyruvate carrier (MPC), which was recently identified in all mammalian cells (54–56). Pyruvate is also linked to the TCA cycle via PC. The cytosolic compartment with direct links through the mitochondria to the TCA cycle also contains citrate, glutamate, and malate. As noted above, the mitochondrial compartment contains the complete TCA cycle with links to the cytosol for citrate and malate. α-Ketoglutarate is linked to glutamate via glutamate dehydrogenase (GLUD) and two aminotransferases, aspartate aminotransferase (GOT), and alanine aminotransferase (GPT).

In order to fit ^13^C time courses of labeled lactate, glutamate and FAs to determine the corresponding metabolic fluxes and the transport parameter for bidirectional lactate transport through the cell membrane, we constructed a BC dynamic model adapted to perfusion bioreactor experiments. In the model, the perfused [1,6-^13^C_2_] glucose and glutamine (labeled in some experiments, see below) are transported from the extracellular medium to the DB-1 cells. Reversible non-steady-state Michaelis–Menten facilitative transport kinetics are assumed to occur through glucose (GLUT family) or glutamine (ASCT2) transporters:

(1) $$J_{i}^{\text{tr}}=\;\frac{J_{\text{tr}}^{\max}\left(\frac{M_{\text{ei}}}{K_{\text{mi}}^{\text{tr}}}-\frac{M_{i}}{K_{\text{mi}}^{\text{tr}}}\right)}{1+\frac{M_{\text{ei}}}{K_{\text{mi}}^{\text{tr}}}+\frac{M_{i}}{K_{\text{mi}}^{\text{tr}}}},$$

where $J_{i}^{\text{tr}}$ is the net transport flux for boundary species *i* between media and cytosol or cytosol and mitochondria; $M_{\text{ei}}$ is the extracellular concentration for species *i*, $J_{\text{tr}}^{\text{max}}$ is the maximal transport rate, and $K_{\text{mi}}^{\text{tr}}$ is the Michaelis–Menten constant for transport. Labeled lactate from the cell is transported into the medium while obeying Michaelis–Menten kinetics through the monocarboxylic acid transporter (MCT family). Facilitated media-cell transport is assumed for glucose, lactate, and glutamine.

Two types of mass balance equations for chemical and isotope variables were expressed mathematically using the mass balance equation for the total metabolite concentration in the medium, cytosol, and mitochondria:

(2) $$\frac{dM_{i}}{\mathrm{dt}}=J_{i}^{\mathrm{tr}}+\sum_{j}\nu_{i}^{\mathrm{pj}}F_{i}^{\mathrm{pj}}-\sum_{k}\nu_{i}^{\mathrm{uk}}F_{i}^{\mathrm{uk}},$$

where *M_i_* is the intracellular concentration of the *i* species, $J_{i}^{\text{tr}}$ is the net transport flux for boundary species *i* between media and cytosol or cytosol and mitochondria, $F_{i}^{\mathrm{pj}}$ and $F_{i}^{\mathrm{uk}}$ are the normalized reaction fluxes that produce (*j*) or utilize (*k*) cellular species *i*, and $\nu_{i}^{\mathrm{pj}}$ and $\nu_{i}^{\mathrm{uk}}$ are the corresponding stoichiometric coefficients. The following general dynamic mass balance equations were used to describe the extracellular boundary metabolites in the medium:

(3) $$\frac{dM_{e}}{\mathrm{dt}}=r_{\mathrm{ie}}\times J_{i}^{\text{tr}},$$

where extracellular species concentration is *M_e_*, and *r_ie_* is the ratio of cell volume to media volume. We also included an additional term for the medium flow rate. Note that we do not assume here that media boundary metabolites are at constant concentration.

### Bonded Cumomer Analysis

One can monitor flux through the biochemical network model by measuring changes in isotopic enrichment of individual carbons in a molecule containing N carbon atoms. This yields a total of N-independent time-courses or steady-state values. However, by considering all the different combinations of labeled and unlabeled carbons, isotopomer models take full advantage of the biochemical information that can be obtained from the NMR spectra, yielding a total of 2*^N^* − 1 independent variables for a molecule with N different carbon atoms. Because not all possible isotopomers are detectable by NMR, a model including all possible isotopomers would be unnecessarily complex. The concept of *bonded cumomers* leads to a reduced number of equations as well as a more simple derivation of these equations compared to a model, including all possible istopomers, while retaining all the NMR measureable isotopomer information.

Let M_{i}_ denote the isotopomer of a metabolite M, where {i} is a set of integers i_1_, i_2_,…, i_n_ indicating the positions of all the labeled carbons. We refer to m_{i}_ as the isotopomer fraction [M_{i}_]/[M] of molecules M labeled exactly at positions i_1_, i_2_…, i_n_. A cumulative isotopomer fraction (or cumomer fraction), noted as p_M{i}_ is by definition, the sum of isotopomer fractions for all isotopomers labeled at least at positions i_1_, i_2_…, i_n_, whatever the label at the other positions. We refer to the size n of the set {i} as the order of the p-function. For example, assuming that M has a total of four carbons, the second order p-function p_M{1,3}_ can be expressed as:

(4) $${\text{p}}_{\text{M}\left\{\text{1},\text{3}\right\}}\;=\;{\text{m}}_{\left\{\text{1},\text{3}\right\}}\;+\;{\text{m}}_{\left\{\text{1},\text{2},\text{3}\right\}}\;+\;{\text{m}}_{\left\{\text{1},\text{3},\text{4}\right\}}\;+\;{\text{m}}_{\left\{\text{1},\text{2},\text{3},\text{4}\right\}}$$

For example, the cumomer fraction p_M{1,3}_ for cumomer M{1,3} is the probability that both the first and third carbon atoms of this molecule are labeled. Shestov et al. (45) have presented a complete exposition of the bonded cumomer formalism. Here, we will present examples of how this formalism can be utilized to efficiently monitor flux through the various metabolic pathways in Figure 1 in various types of perfused cells and in xenografts of these tumor cells in immunosuppressed mice. These examples illustrate the type of information that can be obtained from similar dynamic studies performed on human tumors *in situ* or from extracts of biopsy or surgically excised freeze-clamped specimens of these tumors measured at isotopic steady state.

Isotopomer dynamics of this system together with the initial metabolome state vector *M*_0_ ∈ *R^N^* and fluxome *F_o_* ∈ *R^M^* (subscript “0” refers to baseline steady-state values and reaction rates) are formulated as an initial value problem for ordinary differential equations (ODE) in terms of Bonded Cumomer fractions as the state variables. Metabolite ^13^C cumomer mass balance for parallel monomolecular reactions was expressed in the form:

(5) $$\left[M\right]\frac{d\pi_{\left(i\right)}}{\mathrm{dt}}=\sum_{j}\;F_{j}\pi_{\sigma j\left(i\right)}-\left(\sum_{k}\;F_{k}\right)\pi_{\left(i\right)},$$

where metabolite *M* with pool size [*M*] is downstream of metabolites *S_j_*. While π*_(i)_* and π_σj(i)_ represent the *i*th cumomer fraction of metabolite *M* and metabolite *S_j_* (*i* Bonded Cumomer), respectively. The total outflux Σ*F_k_* balances influx Σ*F_j_*.

Shestov and coworkers (45) have described how isotopomer balance equations have been derived for every metabolite Bonded Cumomer of orders 1, 2, 3 (e.g., glutamate, glutamine, aspartate). This resulted in a set of ~210 differential equations with fine structure multiplets completely described by each metabolite’s Bonded Cumomers of order 1, 2, and 3. Wiechert et al. (43) first proposed the term cumomer, and the concept of cumomer was used by Muzykantov and Shestov (57) in early studies. There are connection matrices between the “Bonded Cumomer” π vectors (which reflect subsets of metabolite isotopomers) and the vectors of “fine structure” multiplets of ^13^C NMR spectra (singlets, doublets, triplets, and quartets of ^13^C-labeled metabolite). Using matrix connection equations, one derives kinetic equations in the form of fine-structure spectroscopic-defined NMR data.

The fitted time-courses for [1,6-^13^C_2_] glucose perfusion were: Glu4Tot (Tot-total), Glu4s, Glu4d34, Glu3Tot, Glu3d, Glu2Tot, Glu2s, Lac3Tot, and acyl methylene (–(CH_2_)_n_–) resonance, for a total of nine curves from which we determined the following free fluxes: melanoma TCA cycle F_TCA_, PC F_PC_, exchange between glutamate and α-ketoglutarate F_X_, anaplerotic exchange and net flux at the level of SucCoA, F_ANA_, mitochondrial malic enzyme (ME2 + ME3) activity, *de novo* FA production, glutaminolysis, aspartate efflux, and three other parameters – Michaelis–Menten lactate transport ${\text{V}}_{\text{max}}^{\text{LAC}}\text{ and }{\text{K}}_{\text{m}}^{\text{LAC}}$ and total cellular lactate concentration. Based on flux balance analysis including non-oxidative glycolytic flux CMRlac and others, we also calculated other fluxes and parameters, including the Warburg parameter (ratio of flux from pyruvate to lactate vs. pyruvate to the MPC). Using the Runge–Kutta fourth-order procedure for stiff systems in terms of Bonded Cumomers, we solved these differential equations to obtain time courses for all possible fine structure ^13^C multiplets of glutamate, glutamine, and aspartate. Minimization of the cost function was performed using Broyden–Fletcher–Goldfarb–Shanno or Simplex algorithms. By verifying that goodness-of-fit values were close to expected theoretical values, we confirmed proper mean-square convergence. Monte Carlo simulations with experimental noise levels were used to calculate parameter errors (58). All numerical procedures were carried out in Matlab (Mathworks, Natick, MA, USA).

Liquid chromatography-mass spectrometry data without detailed flux analysis were utilized to compare fluxes under different conditions (e.g., hyperglycemia vs. euglycemia) since no statistically significant change was observed between the different medium glucose concentrations.

### Metabolic Isotopomer Control Analysis for Bonded Cumomer Models

**Figure 2:**
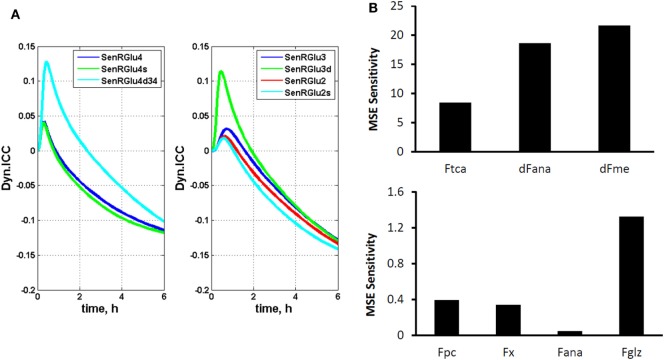
**The dynamic multiplet control coefficients and sensitivity analysis**. **(A)** In the left panel, the dynamic multiplet control coefficients are shown with respect to glutaminolysis flux for the glutamate-C4 total and doublet C4d34 and singlet C4s multiplets and the glutamate-C3 and glutamate-C2 total and their multiplets (right panel) during perfusion with medium containing [1,6-^13^C_2_] glucose and unlabeled glutamine. Data show the time courses of normalized multiplet sensitivities to variation of the glutaminolysis flux. For simulation, the conditions were chosen to optimize extracted fluxes, while glutaminolysis flux was set at 5% of the TCA cycle flux. The glutamate doublet 4d34 is the most sensitive glutamate multiplet to measure glutaminolysis net flux during the first ~1.5 h, and afterwards the 4s singlet becomes more sensitive. The data in the right panel demonstrate the superior sensitivity of the glutamate 3d doublet at the beginning and glutamate 2s singlet after ~1.5 h. **(B)** Total relative mean squared error (MSE) sensitivity function output during perfusion with medium containing [1,6-13C2] glucose and unlabeled glutamine with respect to free fitted fluxes. Upper panel shows the sensitivities to the TCA cycle, anaplerotic net flux at the level of SucCoA and mitochondrial malic enzyme activities; and lower panel shows MSE sensitivities to the pyruvate carboxylase (pc), exchange flux α-Kg ↔ glu (x), exchange flux at the level of SucCoA (ana) and glutaminolysis flux (gls). Note also the significant sensitivities for the fluxes in the upper panel and higher sensitivity for glutaminolysis compared to other fluxes in the lower panel.

Metabolic control analysis (MCA) was originally proposed to quantify sensitivity – i.e., to measure the effect of changes in any parameter of a system on the other variables in that system. This approach was developed independently by two groups in the 1970s (59, 60) and has been limited to systems at steady state. In this approach, the sensitivity of changes in variables, such as metabolite concentrations due to changes in other parameters is quantified by control coefficients. Metabolic control coefficients are used for comparison of many parameters that span several orders of magnitude. A control coefficient is defined as the relative change in the variable per relative change in the parameter, when infinitesimal changes are introduced:

(6) $$C_{p}^{R}={\left(\frac{\partial \text{R}∕R}{\partial \text{p}∕p}\right)}_{\mathrm{ss}}=\;{\left(\frac{\partial \text{lnR}}{\partial \text{lnp}}\right)}_{\mathrm{ss}},$$

where *p* is varied parameter and *R* is a system response, e.g., concentration or flux; the subscript *ss* indicates steady state. Sensitivity analysis measures the robustness of the metabolic model and network topology to variations in parameter values. The robust parameters with greatest influence on model simulation can help to identify critical fluxes and pathways. These fluxes also have less experimental error. Here, we extend the MCA technique to dynamic metabolic flux analysis in which the behavior of interest occurs in the temporal responses. First, we introduce the dynamic isotopomer control coefficient or the sensitivity function:

(7) $$\mathrm{IC}C_{k}^{i}=\left(\frac{\partial \pi_{\left(i\right)}∕\pi_{\left(i\right)}}{\partial F_{k}∕F_{k}}\right),$$

where π*_(i)_* represents the *i*th cumomer fraction of metabolite *M*, and *F_k_* is a certain parameter, such as a flux. By introducing these variables, isotopomer sensitivity equations can be derived by differentiating dynamic bonded cumomer balance equations (Eq. 5) with respect to identified fluxes or other parameters that yield the corresponding initial value Cauchy problem for non-linear ODE. The dynamic sensitivity equations determine the time evolution of isotopomer control coefficients. These consist of a large number of equations that must be solved simultaneously with the initial system (1) resulting in double the number of differential equations. These characteristics of the output sensitivity matrix with respect to the flux vector ***F*** and other parameters provide a framework for analysis of isotopomer model robustness. By simulating the dynamic time course of sensitivity functions, one identifies the fluxes most sensitive to a particular bonded cumomer of a metabolite and ^13^C fine structure multiplet. Figure 2A displays calculated values of those dynamic isotopomer control coefficients (or relative sensitivity functions).

We also computed the sensitivity of the mean squared difference between experimental and corresponding model output (i.e., error) to finite changes in parameter values. The sensitivity to each parameter was calculated as the relative change in mean squared error (MSE) due to 5% change in the given parameter value:

(8) $$S_{i}=\;\frac{\max\left|\left(E\left(p_{i}\pm 0.05p_{i}\right)-E\left(p_{i}\right)\right)\right|}{\frac{0.05p_{i}}{p_{i}}*E\left(p_{i}\right)},$$

where *E* represents the minimum mean squared difference between model simulation and experimental data. These sensitivity values represent the degree to which the theoretical curves are sensitive to the value of individual parameters. High values of the sensitivity parameter *S_i_* indicate that changing a parameter *p_i_* results in a significant change in the simulated curves and in the MSE *E*.

Figure 2B shows calculated MSE sensitivities for several free fitted fluxes.

### Applications of Metabolomics to Melanoma and Lymphoma

To illustrate the application of these techniques to studies of human cancer, we will describe studies of human melanoma models grown in culture, as perfused tumor cells grown on solid microcarrier beads and as xenografts in immunosuppressed mice. The cell studies will be utilized to validate the bonded cumomer model and to estimate the contributions of glycolytic metabolism and mitochondrial metabolism to ATP production under hyperglycemic (26 mM) and euglycemic (5 mM) conditions. Effects of euglycemia and hyperglycemia on glutamine metabolism, flux through the pentose shunt and FA metabolic pathways will also be evaluated using both ^13^C MRS and bonded cumomer analysis as well as LC–MS and fragmented cumomer analysis.

To demonstrate how ^13^C MRS and LC–MS can be used to detect response to signal-transduction inhibitors, we will present data on the effect of rapamycin, a well known inhibitor of the mTOR pathway, on DLCL2 human diffuse large B-cell NHL cells grown in culture as perfused cells immobilized by encasement in agarose beads and as xenografts in immunosuppressed mice. We will demonstrate that response to this inhibitor can also be monitored by ^1^H MRS lactate imaging in cells and in xenografts. Ultimately, the goal will be to translate these methods into the clinic to manage human NHL patients. Preliminary studies leading to this approach have been published (15).

## Materials and Methods

### Cell Culture

The human DB-1 melanoma cell line was derived by David Berd from a lymph node metastasis of one of his patients (Thomas Jefferson University Hospital, Philadelphia, PA, USA) before administration of any treatment. Cells were cultured from the tumor and cryopreserved after the 16th passage. Monoclonal antibodies were used to confirm cell surface antigens (61). DB-1 cells were grown as monolayers for routine culture at 37°C in 5% CO_2_ in α-MEM medium supplemented with 10% fetal bovine serum, 2 mM glutamine, 26 mM glucose, and 1% (v/v) non-essential AAs and 10 mM HEPES buffer. In tissue culture flasks, the doubling time was 48 h (50). The V600E BRAF mutation is expressed by the DB-1 cells.

### Cell Perfusion and NMR Spectroscopy

For NMR studies of perfused tumor cells, DB-1 cells were cultured in DMEM with 1% non-essential AAs (Invitrogen, Grand Island, NY, USA), 10% fetal bovine serum, 10 mM HEPES, 4 mM glutamine, and 26 mM glucose (complete DMEM). Approximately 5 × 10^8^ cells were grown on the surface of non-porous microcarriers that had a mean diameter of 170 microns (Solohill, Ann Arbor, MI, USA). These microcarriers were coated with either collagen or ProNectinF^®^ to enhance cell attachment and proliferation and tightly packed inside a 20 mm NMR tube in which they were perfused continuously in the open bioreactor system. Mancuso et al. (62) have published a detailed description of the perfusion system, including flow rates, cell adhesion procedures, etc.

The method of Bental et al. (63) was used to immobilize lymphoma cells (DLCL2) by encapsulation into agarose beads. In brief, we thoroughly mixed 1.5 ml of ~5 × 10^8^ cells in culture medium with an equal volume of the low-temperature-gelling agarose at 37°C and magnetically stirred the mixture after addition of 3 ml paraffin oil at 37°C generating the cell-encapsulated spherical beads with 100–200 μm diameters. The beads were cooled by continuous stirring, and the oil was removed by centrifugation. The encapsulated cells were then loaded into a 10 mm NMR tube connected to the perfusion system.

Medium was circulated through the microcarriers or agarose gel at a flow rate of 12 ml/min with a peristaltic pump (Masterflex, Cole Parmer, Chicago, IL, USA). A gas-exchange module consisting of a silicone membrane (thin-wall silicone tubing) was situated before the perfusion chamber along the medium flow path for removal of carbon dioxide and addition of oxygen. The oxygen level was measured continuously with a polarographic oxygen probe (Mettler-Toledo, Columbus, OH, USA) situated at the inlet port to the perfusion chamber and maintained at a steady-state pressure near air saturation. The medium was then warmed to 40°C, and a second polarographic oxygen probe was used to detect the oxygen level of the medium coming out of the NMR tube permitting the oxygen consumption rate of the culture to be monitored continuously from the decrease in pO_2_. A pH probe (Mettler-Toledo, Columbus, OH, USA) was inserted downstream of the outlet oxygen probe. Adjustment of the level of CO_2_ in the gas-exchange module was used to modify/maintain the pH of the medium. The temperature of the medium entering the NMR tube was monitored with a thermocouple at the exit port of the perfusion chamber and was fine-tuned with a microstat-controller and an electrical resistance heater to 37.0 ± 0.2°C. The glucose concentration in the recirculating medium was maintained at a constant level (clamped) by continuously feeding fresh medium typically at a flow rate of 24 ml/h and removing depleted medium from a recirculation bottle while maintaining the total volume of recirculating medium at 120 ml. During ^13^C experiments, 32 mM [1,6-^13^C_2_] glucose was fed into the system, and the recirculating glucose level was clamped at 26 mM by adjusting the feed rate.

^13^C NMR spectra were acquired with standard ^1^H decoupled NOE ^13^C acquisition on a 9.4 T/89 mm vertical bore Varian spectrometer (Varian Inc., Palo Alto, CA, USA) with acquisition parameters: 60° pulse angle, 1.2 s repetition rate, 25,000 Hz spectral width, 16,384 points, and 750 transients per spectrum. Free-induction decays were apodized by exponential multiplication (2 Hz) for signal to noise enhancement and peak areas were measured with Nuts NMR (Acorn NMR, Fremont, CA, USA) software. The number of cells in the NMR tube was determined from the total NTP level measured by ^31^P NMR as described previously (64) using the following spectral parameters: 60° pulse angle, 1 s repetition rate, 15,000 Hz, 1,200 transients.

Melanoma cells were initially studied under normoxic hyperglycemic (26 mM glucose) conditions to enhance lactic acidosis (65, 66) and subsequently under normoxic euglycemia (5 mM glucose) to more closely simulate *in vivo* conditions. The effect of these changes in glucose concentration on flux through various pathways of tumor metabolism was determined over a ~6 h time course during which the kinetics of labeling of lactate, glutamate and other metabolites was monitored. The estimated TCA cycle flux was compared to the oxygen consumption flux to test the accuracy of the calculations.

### *In vivo* animal studies

Immunosuppressed mice were used for *in vivo* studies. Tumors were developed by subcutaneous implantation of tumor cells to the flanks of nude mice. NMR studies on mice were performed using a home-built ^13^C/^1^H dual-tuned probe at 9.4 T (Varian Inc.). All animal studies were approved by the Institutional Animal Care and Use Committee (IACUC) of the University of Pennsylvania, and performed in accordance with its regulatory standards.

### Statistical Analysis

Student’s *t*-test was used to calculate *p*-values. *P*-values <0.05 were considered significant.

## Results and Discussion

### Melanoma Studies

Determination of the relative levels of glycolytic and oxidative metabolism of specific tumors plays an important role in development of strategies for treating neoplasms and for choosing appropriate methods to detect tumor response to specific therapies. Development of a non-invasive method for quantifying the fraction of ATP production by tumors that occurs by glycolytic and mitochondrial metabolism would be very useful in the management of cancer patients and tailor-fitting of therapeutic regimens to their individual needs. In addition, various other pathways of tumor metabolism play critical roles in tumor proliferation by providing essential precursors for cell replication. We, therefore, sought to develop a method for quantitating tumor intermediary metabolism in cells with appropriate extension to animal tumor models and eventually to human cancer patients.

G lucose in DB-1 Cells]

### Metabolic Fate of [1,6-^13^C_2_] Glucose in DB-1 Cells

**Figure 3:**
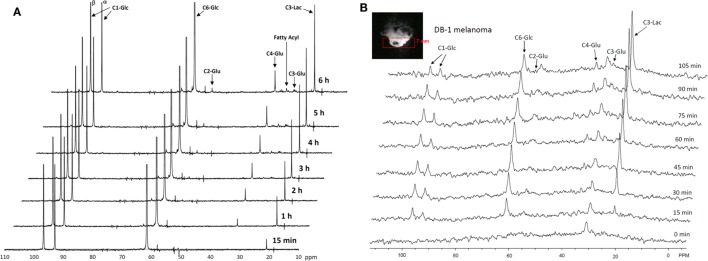
**Time course ^13^C NMR spectra of perfused DB-1 melanoma cells in a bioreactor (A), and of a DB-1 melanoma xenograft (B)**. Glu, glutamate; Lac, lactate; Glc, glucose; std, ethanol in capillary; FA, –CH_2_– groups in long chain fatty acids. [1,6-^13^C_2_] glucose was used as the substrate for ^13^C-labeling.

**Figure 4:**
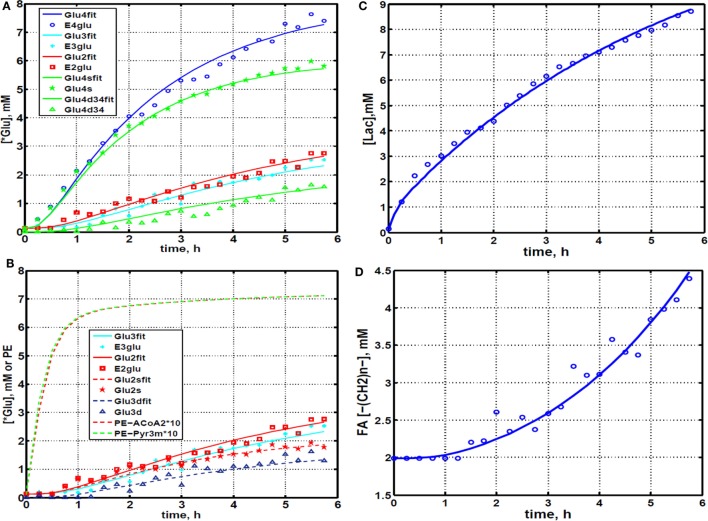
**Time courses for labeling of glutamate, lactate, and fatty acyl resonances during perfusion with medium containing [1,6-^13^C_2_] glucose and unlabeled glutamine**. **(A)** Time course fits for labeling of glutamate (indicated with “fit” ending) at positions 4, 3, and 2. Glu4, ^13^C at glutamate-C4; Glu4s, singlet at glutamate-C4; Glu4d34, doublet at glutamate-C4. Glu2, 13C enrichment at glutamate-C2; Glu3, 13C at glutamate-C3. “fit” ending stands for the model fit and no-ending reflects experimental values. **(B)** Time courses for labeling glutamate at positions 2 and 3. Fitted curves labeled with ending “fit” are Glu2 and Glu3 total; Glu2s, singlet at glutamate-2; Glu3s, singlet at glutamate-3; Glu3d, doublet at glutamate-3. PE stands for positional enrichment – this is predicted mitochondrial enrichment of acetyl-CoA at position C2 and mitochondrial Pyr at position C3 multiplied by factor 10. Curves are fit, and points are experimental data. **(C,D)** Experimental data and least-squares fit of time course for labeling of lactate-C3 and fatty acyl methylene (–(CH_2_)_n_–) resonance in proliferating DB-1 melanoma cells. **(C)** Time course for labeling of total lactate-C3 (medium + intracellular). **(D)** *De novo* lipogenesis in melanoma cells. Time course for labeling of fatty acyl methylene (–(CH_2_)_n_–) resonance. [This research was originally published in Journal of Biological Chemistry. Shestov et al. (14). © the American Society for Biochemistry and Molecular Biology.]

The entire metabolic network that is included in the current analysis is depicted in Figure 1. Figures 3A and 4 summarized ^13^C labeling kinetics of DB-1 melanoma cells grown at a constant glucose level of 26 mM. Dynamic ^13^C NMR spectra obtained during the experiment appear in Figure 3A. We detected resonances of C3 lactate, C2, C3, and C4 glutamate, C2 and C3 of aspartate, and fatty acyl carbons. Under hyperglycemic conditions (26 mM glucose), lactate labeled at C3 (20.9 ppm) appeared in the spectrum within the first few minutes (Figures 3A and 4C). Levels of this metabolite increased throughout the experiment but did not reach saturation. Lactate resonance intensity was the combined result of the biological rate of lactate formation and the rates of washout of lactate due to the continuous addition of fresh medium and withdrawal of spent medium from the perfusion system. Fifteen minutes after introduction of labeled glucose, isotopic ^13^C incorporation in C4 of glutamate (34.3 ppm) was detected; Figure 4A shows the full-time course for labeling results for this resonance. The concentration of C4-labeled glutamate increased linearly for ~1 h. Satellites were subsequently detected indicating the presence of ^13^C-^13^C coupling between glutamate-C4 and glutamate-C3 (1). The rate of labeling of glutamate exclusively on C4 (producing a singlet peak) gradually slowed and approached steady state after approximately 6 h. The level of coupled glutamate labeled at both C3 and C4 continued to increase steadily throughout the experiment. Labeling of the glutamate-C3 resonance at 28.8 ppm is displayed in Figures 4A,B. ^13^C labeling of this carbon increased linearly but did not reach saturation. Approximately, two-thirds of the C3 glutamate carbon was coupled (predominantly to glutamate-C4). Within 1 h, glutamate-C2 labeling was detected (Figures 4A,B). However, only a singlet was observed for the first 3 h, unlike glutamate-C3 which exhibited multiplet structure. Subsequently, a doublet appeared due to the presence of glutamate-C2,3 labeling after carbon 1 was removed from glutamate-C3,4 during the third turn of the TCA cycle (1).

Labeling was also observed in long chain FAs at approximately 30 ppm, indicating that DB-1 melanoma cells produce lipid *de novo* from glucose (see Figure 4D), consistent with results for other cancer cell lines grown in culture (67). Low levels of labeling of aspartate-C2 and C3 were also observed. Labeling of alanine was not detected, probably due to the low total concentration of this AA; this result is unusual since most cancer cell lines produce ^13^C-labeled alanine from glucose (62, 67). Resonances were observed at approximately 65 ppm, a spectral region associated with the glycerol backbone of phospholipids in triglycerides as well as other glycolytic intermediates.

### Quantifying Bionetwork Fluxes

We utilized Bonded Cumomer analysis to fit ^13^C NMR DB-1 melanoma data acquired during continuous perfusion of cells with [1,6-^13^C_2_] glucose in the bioreactor system. Isotopomer time course data were converted to ^13^C-metabolite concentration curves (Figure 4). Only those multiplets with end-point concentrations >0.5 mM were used in the fitting procedure in order to minimize the bias introduced by using multiplets with low signal to noise ratio. Since the noise level of the remaining experimental multiplets was expected to be high (not shown), these resonances were used only as reference points for visually examining the fit predicted for low intensity curves to experimental data but not for calculating the goodness of fit of these metabolite resonances. Four types of measurements were used to quantify tumor central metabolic fluxes: three sets of time courses of (1) glutamate isotopomers (multiplets), (2) total lactate-C3 labeling, (3) acyl groups of FA resonances determined by NMR *in situ*, and (4) steady-state ^13^C NMR lactate isotopomers. The following time courses were utilized in the curve-fitting procedure: Glu4Tot (Total: Tot), Glu4s, Glu4d34, Glu3Tot, Glu3d, Glu2Tot, Glu2s, Lac3Tot, and acyl methylene (–(CH_2_)_n_–) resonance, yielding a total of nine curves. Estimation of the model-free parameters was achieved by fitting labeling patterns of glutamate, lactate, and FAs acyl methylene groups. Table 1 and the flux map in Figure 1 summarized the fluxes determined by the fitting procedure. Monte Carlo simulations were used to estimate errors in parameters included in Table 1.

**Table 1:** **Extracted fluxes using bonded cumomer modeling**.

Reaction	Flux (mmol/L-cell/h)^a^
Pentose phosphate pathway (oxidative branch), net PPP F_pppOx_	3.3 ± 72%
Transketolase 1 (exchange) relative to glycolysis, TK1	21 ± 56%
Transketolase 2 (exchange) relative to glycolysis, TK2	0.5 ± 90%
Transaldolase (exchange) relative to glycolysis, TA	44 ± 64%
Glucogenic amino acids contribution to pyruvate, F_gaa_	5.0 ± 20%
TCA cycle rate, F_tca_ (isocitrate → α-Kg)	10.9 ± 8%
Exchange, Mal-Asp shuttle, F_x_ (reversible)	11.3 ± 19%
Pyruvate carboxylase Flux, F_pc_	0.6 ± 51%
Anaplerotic exchange flux at SucCoA, F_xana_	3.0 ± 15%
Net Anaplerotic flux to the TCA cycle, dF_ana_	4.3 ± 15%
Flux of ketogenic AA and FA to Acetyl-CoA formation, F_kaa_	0.1 ± 19%
Glutaminolysis Flux, F_gls_	0.05 ± 60%
Pyruvate dehydrogenase Flux, F_pdh_	10.8 ± 16%
Citrate synthase flux, F_cs_	11.5 ± 14%
Net fumarase activity, Fum →Mal, F_fum_	15.3 ± 16%
Fatty acids synthesis, F_fas_	0.59 ± 15%
Mitochondrial pyruvate transport, F_mpc_	7.1 ± 23%
Malic enzyme (mitochondrion), ME2 + ME3, F_mem_	4.3 ± 12%
Malic enzyme (cytosolic), ME1, F_mec_	0.6 ± 15%
Aspartate efflux, dF_xasp_	0.06 ± 45%
Production Flux of ATP, glycolytic, Warburg, F_atpCyt_	150 ± 13%
Production Flux of ATP, mitochondrial, oxphos, F_atpOx_	154 ± 12%
Warburg parameter (net LDH/MPC flux ratio)	21 ± 24% (unitless)
Combined glycolysis and PPP flux from G6P to pyruvate	152.1 ± 12%
Glutamine consumption flux (for protein, nucleotides, amino acids, glutathione, etc)	4.9 ± 10%^b^
Net LDH activity, Pyr → Lac, F_ldh_	150 ± 12%
Lactate transport, V_maxlac_	395 ± 12%
Lactate transport, Michaelis–Menten parameter, K_m_	3.5 mM ± 70%

*^a^Calculated rates of flux are expressed in millimoles per liter-cell per hour with relative SDs in percent for pathways shown in Figure 1. Warburg parameter is the ratio of the net flux through lactate dehydrogenase (LDH) to the mitochondrial pyruvate carrier influx (MPC)*.

*^b^SD was estimated based on experimental measurements of glutamine consumption*.

*This research was originally published in Journal of Biological Chemistry. Shestov et al. (14). © the American Society for Biochemistry and Molecular Biology*.

### Model Validation

To validate the bonded cumomer network model, we compared parameters inferred from the model with experimentally determined values. We measured the O_2_ consumption rate (MRO_2_) with polarographic oxygen probes during the perfusion experiment. The fluxes obtained by fitting of dynamic ^13^C data produced good agreement between the experimentally observed oxygen consumption rate, 33 mmol/L-cell/h, and the calculated MRO_2_, 32 mmol/L-cell/h (see Tables 2 and 3). The experimental total glutamate concentration was 8.6 mM that was in good agreement with the calculated glutamate pool size of 9.2 mM. The measured glucose consumption rate (MRglc) was 90 mmol/L-cell/h, that compared favorably with the predicted MRglc of 81 mmol/L-cell/h. The measured lactate production flux (CMRlac) was 155 mmol/L-cell/h, whereas the predicted CMRlac was 150 mmol/L-cell/h. We note that we did not use experimentally measured fluxes as constraints in our Bonded Cumomer metabolic flux analysis.

**Table 2:** **Experimental data compared to calculated values determined by dynamic bonded cumomer modeling (mmol/L-cell/h)**.

Reaction	Exp. Flux (mmol/L-cell/h)	Calculated flux (mmol/L-cell/h)
Oxygen consumption MRO_2_	36	32
Glucose uptake	91.0	85
Pyruvate to lactate	155.0	150
Glutamine uptake	5.0	–^a^
Glutamate concentration	8.6 mM	9.2 mM

*^a^The flux was not estimated, as glutamine is consumed in numerous reactions we did not study here. This research was originally published in Journal of Biological Chemistry. Shestov et al. (14). The American Society for Biochemistry and Molecular Biology*.

**Table 3:** **Experimental glucose and glutamine consumption fluxes under euglycemia (6.4 mM) and hyperglycemic conditions (23.2 mM in medium)**.

	Glutamine flux	Glucose flux
Euglycemia (6.4 mM)	0.064	0.86
Hyperglycemia (23.2 mM)	0.035	0.79

*Glutamine [Gln] = 3.0 mM at low glucose and 3.4 mM at high glucose. Fglc, glucose consumption flux (mmol/10^9^cells/h) and Fgln, glutamine consumption (mmol/10^9^cells/h). Flux ratios were Fglc/Fgln = 22.4 at high glucose and Fglc/Fgln = 13.5 at euglycemia*.

*This research was originally published in Journal of Biological Chemistry. Shestov et al. (14). © The American Society for Biochemistry and Molecular Biology*.

Tables 2 and 3 also compare glucose and glutamine uptake under euglycemic (6 mM glucose) and hyperglycemic (23 mM) conditions. Under euglycemic conditions, glutamine influx approximately doubles, whereas glucose consumption remains essentially unchanged.

Finally, we were able to generate a flux map that was included with the bionetwork in Figure 1. This figure shows the extracted metabolic fluxes with the predicted oxygen consumption rate MRO_2_. Numbers indicate fluxes in mmol/L-cell/h. See Table 1 for definitions and values of the derived fluxes; Table 2 summarizes the predicted and calculated metabolic rates derived from these data. The excellent agreement between model-predicted and experimental values validates the model.

### Quantifying ATP Production Routes

The predicted fluxes were used to calculate contributions of glycolysis and oxidative phosphorylation (oxphos) to ATP production. We assumed that the oxidative phosphorylation P/O ratio equals 1.5 for FADH_2_ and 2.5 for NADH oxidation. To assess the contribution of glucose, FAs and glucogenic AAs and glutamine to generating reducing power, we calculated the fluxes for each of these metabolites. For each of the above substrates, we calculated the NADH/FADH_2_ reaction flux based on the different labeling patterns that we were able to track both from the labeled and unlabeled C atoms. The combined cytosolic ATP production flux was 150 mmol/L-cell/h, and the mitochondrial compartment produced 154 mmol/L-cell/h due to oxidative phosphorylation. The results are presented in Table 1.

Consequently, about 50% of the energy of this tumor model comes from oxidative metabolism, whereas many authors have suggested that tumors derive most of their energy from aerobic glycolysis. The glycolytic contribution will, no doubt, increase *in vivo* where tumors are often located in regions of hypoxia, which produce a Pasteur effect, and, hence, a shift toward glycolytic metabolism; however, it is likely that many tumors like melanomas will be strongly dependent on oxidative metabolism for their energy source, particularly in tumors that express the V600E BRAF mutation, about 50% of human melanomas. (68) High fluxes of pyruvate mitochondrial transport (~70% of the TCA cycle activity) and mitochondrial ATP production are promoted by melanoma associated BRAF mutation. Recent work (69) has demonstrated that BRAF V600E mutated melanoma cells exhibit increased pyruvate entry through the activated PDH. PDH flux (~106% of the TCA flux) is regulated by phosphorylation/dephosphorylation mediated by PDH kinase (PDK) and PDH phosphatase (PDP). The oncogene BRAF V600E causes concerted activation of PDH complex by downregulation of PDK1 expression and upregulation of PDP2 thereby promoting oxidative pyruvate utilization and leading to increased cellular respiration.

### Pentose Phosphate Pathway

**Figure 5:**
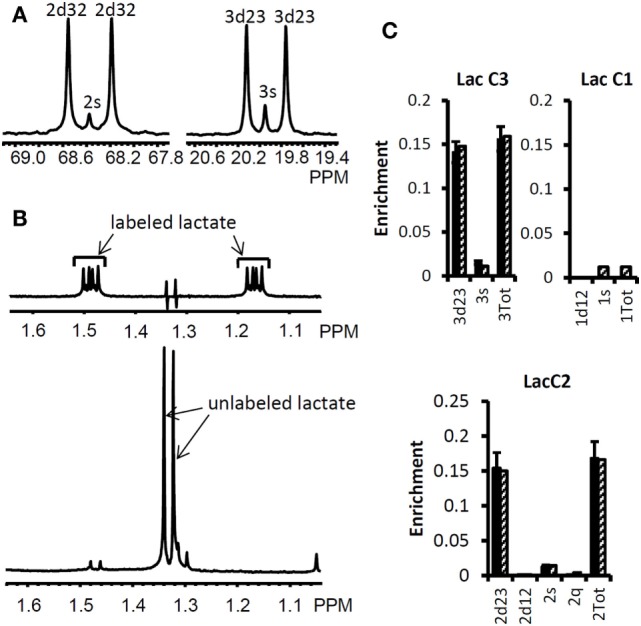
**High-resolution ^13^C and ^1^H NMR spectra of lactate from DB-1 cells incubated with [1,2-^13^C_2_] glucose and experimental lactate multiplets vs. fit**. **(A)** ^13^C NMR spectra. The left panel shows the NMR spectrum at the chemical shifts around lactate C2, and the right panel shows the spectrum around lactate C3. 2s, lactate singlet at C2 position; 2d32, lactate C2 doublet with carbon coupling between the carbon 2 and 3 positions; 3s, lactate singlet from the C3 position; 3d23, lactate C3 doublet with carbon coupling between the carbon 2 and 3 positions. **(B)** Proton- observed carbon-edited (POCE) ^1^H NMR spectra. The upper panel shows the labeled lactate and the lower panel shows the unlabeled lactate. **(C)** Experimental multiplet fraction (left bars, black) vs. fitting (right bars, patterned) for lactate C3 and C2, and the predicted lactate C1 multiplets, based on the evaluated oxidative and non-oxidative branches of the PPP. Error bars indicate SDs. The extracted oxidative PPP flux and the relative exchange fluxes for transketolases and aldolase are presented in Table 1. [This research was originally published in Journal of Biological Chemistry. Shestov et al. (14). © the American Society for Biochemistry and Molecular Biology.]

We used the Bonded Cumomer flux model to fit ^13^C NMR steady-state data acquired after 8 h incubation of DB-1 cells with [1,2-^13^C_2_]glucose tracer. The fitted steady-state data were Lac3Tot, Lac2Tot, Lac3s, Lac3d23, Lac2s, and Lac2d23 multiplets. All the experimental ^13^C NMR spectra and the results of fitting are shown in the Figure 5. The extracted fluxes relative to glycolytic flux are presented in Table 1. Flux SDs were calculated by Monte Carlo calculations. Flux through the oxidative PPP was estimated to be 3.6% of the glycolytic rate. Given the extensive experimental data that have been obtained for the lactate ^13^C NMR multiplets, we were also able to estimate non-oxidative exchange fluxes (the smallest rate for reversible reaction) of the classical PPP (Figure 1), which are presented in Table 1. Transketolase 1 and transaldolase exchange fluxes in the non-Ox PPP branch were consequently relatively high at 21 and 44%, respectively, and transketolase 2 activity was close to 0. The flux SDs (Table 1) demonstrate that all fluxes through PPP were resolved with relatively poor resolution when using only ^13^C NMR isotopomers of lactate as a reporter molecule. NADPH production flux via the oxidative PPP pathway was estimated to be 6.6 mmol/L-cell/h.

### Quantifying Glutaminolysis Flux

Our recent focus has turned to refinement of the details of glutaminolysis because of its critical role in the metabolism of many malignancies. For example, one glutamine molecule could produce up to 22.5 ATP molecules. Therefore, even a small amount of glutaminolysis can have a major effect on the bioenergetics of cancer cells. In DB-1 melanoma under hyperglycemic conditions, glutamine uptake flux ranged up to 50% of the TCA cycle flux. Under euglycemic conditions, glutamine uptake doubled reaching approximately the same level as TCA cycle flux. Glutamine uptake can lead to protein synthesis, glutathione synthesis, or nucleotide synthesis as well as to ATP production by the glutaminolysis pathway (see below) or to cytosolic reductive carboxylation (RC) driven by glutaminase 1 (GLS; it is considered to be present both in mitochondria and cytosol) or other glutamine–glutamate conversion enzymes in cytosol and isocitrate dehydrogenase 1 (IDH1) (70). It was, therefore, important to refine the metabolic analysis of DB-1 melanoma to accurately assess the contributions of the glutaminolysis pathway in these tumor cells (See Figure 1). In order to quantify the glutaminolysis contribution to energy production, we modified the bionetwork simultaneously allowing net glutamine influx into mitochondria through glutaminase 1 and 2 (GLS and GLS2) (Figure 1).

“Classical” glutaminolysis includes uptake of glutamine by the cell, transport to mitochondria, conversion to glutamate through mitochondrial glutaminase 1 or 2 (GLS and GLS2), and finally entry into the TCA cycle to α-ketoglutarate via GLUD1 and GLUD2 or via mitochondrial aminotransferases (GPT2 and GOT2). Alternatively, glutaminolysis could partially occur in the cytosol by producing glutamate or α-ketoglutarate from glutamine and eventually by entry of those metabolites in to the TCA cycle via α-ketoglutarate. Further flux in the TCA cycle then moves to malate where malate–pyruvate cycling occurs (Figure 1). There are two pathways for pyruvate-malate cycling. One pathway involves conversion of malate via mitochondrial malic enzyme (ME2) to pyruvate; it then returns to oxaloacetate via PC, or it can go from pyruvate to acetyl-CoA leading to citrate. The second pyruvate cycling pathway goes from malate in the mitochondria through a transporter into the cytosol and from there to cytosolic pyruvate via cytosolic malic enzyme (ME1). The cytosolic pyruvate can return to the mitochondria through the MPC or it can be converted to lactate via LDH or to alanine via alanine aminotransferase (GPT1). The BC approach can distinguish between these pyruvate–malate pathways or their combinations under real conditions (Figure 1).

We continue the analysis of data of DB-1 melanoma cells treated with [1,6-^13^C_2_]glucose as the tracer with respect to sensitivities of the experimental multiplets to glutaminolysis. Dynamic isotopomer control analysis (ICA) indicates that the curves resulting from fitting of the C4-labeled glutamate multiplets, particularly the glutamate 3,4 doublet-glu4d34 resulting from simultaneous labeling of C3 and C4 (during the second TCA cycle turn) and the singlet resulting from glutamate labeling exclusively at C4 (during the first turn of the TCA cycle) are very sensitive to flux through the glutaminolysis pathway even when only natural abundance medium glutamine has been used. See for example Figure 2A, where we display the dynamic multiplet control coefficients with respect to glutaminolysis activity for the glutamate C4 total and the glutamate C4 singlet and 3,4 doublet-4d34, and for the glutamate C3 and C3d doublet and glutamate-C2 and C2s singlet. This figure clearly indicates that glutamate labeling kinetics is sufficiently sensitive to glutaminolysis flux and that there is no need to purchase or synthesize exotically labeled glutamine (with ^13^C, ^15^N, or ^2^H labeling) to evaluate glutaminolysis. Control simulation was based on the optimal set of extracted fluxes, and glutaminolysis flux was set at 5% of the TCA cycle flux. The plotted dynamic glutamate multiplet control coefficients indicate that the glutamate doublet 4d34 is the most sensitive glutamate multiplet with respect to glutaminolysis net flux during the first ~1.5 h when the sensitivity of the RGlu4d34 doublet is positive (positive mode) and afterwards when the RGlu4s singlet 4s becomes most sensitive in the negative operation mode. The data in the right panel of Figure 2A demonstrate the superiority of the sensitivity to glutaminolysis flux of the glutamate 3d doubled at the beginning and glutamate 2s singlet during the time course after ~1.5 h.

We then evaluated the contribution of glutaminolysis flux to overall energy production of DB-1 melanoma cells. We found that for this tumor line, glutaminolysis flux through the truncated TCA cycle and via pyruvate–malate cycling by malic enzyme ME2 is essentially 0. This conclusion resulted directly from the ability of the model to detect glutaminolysis flux by fitting of data on the glutamate multiplets (see above). To further confirm this conclusion, we forced the model to accommodate glutaminolysis flux between 5 and 20% of the TCA cycle flux. Invariably, this grossly deteriorated the goodness-of-fit of the isotope kinetic data to the model (results not shown).

Even though glutaminolysis does not contribute substantially to mitochondrial ATP production, there is still substantial mitochondrial malic enzyme flux (combined activities of NADH producing ME2 and NADPH producing ME3), which is equal to approximately 40% of the TCA flux (see Table 1).

There has been considerable interest in glutamine metabolism as a key source of tumor energy production linked to c-myc expression (71–75). Our model of tumor metabolism (Figure 1) contains a specific glutamine transporter on the cell membrane. The classical model of glutaminolysis includes an additional specific glutamine transporter on the mitochondrial membrane that leads to mitochondrial glutaminase (GLS2 or GLS), which converts glutamine to glutamate, which is then converted to α-ketoglutarate by enzymes, such as GLUD1 and GLUD2. Two additional transaminases can carry out the glutamate to α-ketoglutarate exchange, aspartate aminotransferase, and alanine aminotransferase. The α-ketoglutarate then produces reducing equivalents via the oxidative branch of the TCA cycle leading to ATP production by oxidative phosphorylation. Various pathways, including the malate-aspartate shuttle, PC, malic enzyme, and phosphoenolpyruvate carboxykinase (PEPCK1 and 2) all contribute to metabolism of the glutamine carbon atoms. As previously noted, glutamine metabolism can lead to production of 22.5 molecules of ATP per glutamine molecule. In the case of DB-1 melanoma cells under hyperglycemic conditions, glutamine influx constituted about 50% of the TCA cycle flux, with about double this level of glutamine uptake under euglycemic conditions (Tables 2 and 3). However, the transaminase reactions are reversible and may exhibit high exchange fluxes in both directions with very little net glutamine uptake by the TCA cycle and nearly no energy production from the glutaminolysis pathway in DB-1 cells. It is important to note that because carbon atoms of glutamate originating from glucose will be isotopically labeled (first on C4 position and then on C2 and C3, etc.), whereas carbons coming from glutamine will be unlabeled, it was possible to distinguish the two pools of carbon atoms originating from these two substrates. The data fitting clearly indicated that the net flux from glutamine being metabolized via the TCA cycle was very small.

We also conducted a sensitivity analysis to show that using [1,6-^13^C_2_] glucose tracer alone is enough to reliably calculate glutaminolysis flux in malignant cells and tissues. Figure 2A shows that the glutamate-C4 doublets due to C3–C4 coupling (glu4d34) is most sensitive to glutaminolysis and all experimental glutamate multiplets, including total positional enrichments at glutamate C4, C3, and C2 are sensitive to the net flux of unlabeled glutamine entering the TCA cycle. Fitting analysis indicated that there was little net influx from glutamine into the TCA cycle. Note also that this conclusion can also be reached from ^13^C NMR data without the use of labeled glutamine.

**Figure 6:**
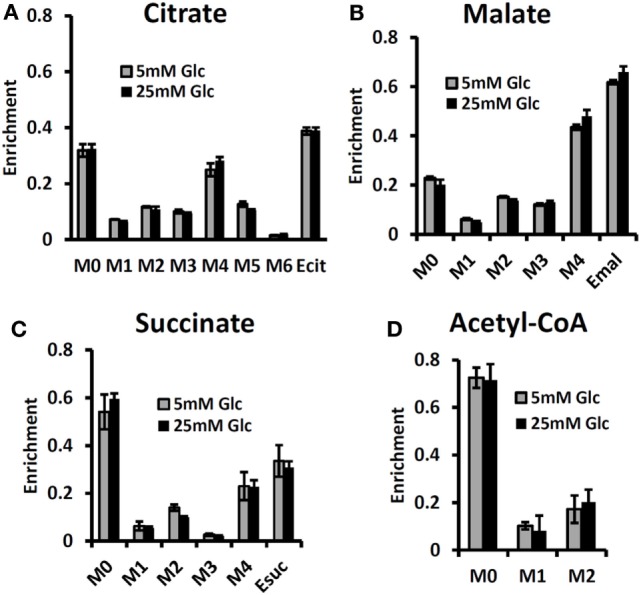
**Mass-isotopomer distribution (MID) for the TCA cycle intermediate and acetyl-CoA measured at hyperglycemic and euglycemic conditions during [U-^13^C_5_] glutamine labeling**. MID of **(A)** citrate, **(B)** malate, **(C)** succinate, and **(D)** acetyl-CoA. The figure clearly demonstrates that there are no significant differences in labeling patterns of key metabolites (*p*-value was always >0.05) between 5 mM glucose and 25 mM medium glucose conditions. [This research was originally published in Journal of Biological Chemistry. Shestov et al. (14). © the American Society for Biochemistry and Molecular Biology.]

By contrast, some investigators utilizing mass spectrometry have equated glutamine uptake minus glutamate release with net glutaminolysis flux (76). In the present case, the net influx of glutamine is ~5 mmol/L-cell/h, which, if it were equated with glutaminolysis flux, would have been interpreted as 113 mmol/L-cell/h of ATP per h or about 75% of the glycolytic production of ATP. Under euglycemic conditions, which produce about twice the level of glutamine uptake, such an analysis would have attributed about 50% higher ATP production to glutaminolysis rather than to glycolysis, and most of the tumor energy production would have been attributed to glutaminolysis. This is incorrect in our case; in reality the net glutaminolysis flux under hyperglycemic conditions is ~0.1 mmol/L-cell/h or ~2 mmol ATP/L-cell/h or ~1% of the glycolytic ATP production rate. Bonded Cumomer analysis provides a method for making this crucial distinction. Our labeled LC-MS data for the TCA cycle intermediates with [U-^13^C_5_, U-^15^N_2_] glutamine confirmed that no glutaminolysis occurred under euglycemic conditions vs. hyperglycemia (Figure 6). The absence of change in glutaminolysis or RC flux is evident from similar mass-isotopomer distribution (MID) of several key metabolites. Our finding of nearly zero glutaminolysis flux contradicts the view of Scott et al. (77), who, on the basis of glutamine uptake and label appearance in some TCA cycle metabolites, concluded that glutamine was utilized as an anaplerotic substrate of several human melanoma lines. However, these authors failed to conduct a rigorous metabolic modeling analysis to measure flux through the TCA cycle. Consequently, they were unable to distinguish between actual flux through the cycle via glutaminolysis and exchange labeling between α-ketoglutarate and glutamate without any net flux. In addition, glutamine could be used for many other metabolic processes besides energy production, e.g., protein and nucleotide synthesis. If the net uptake of glutamine is 5 mmol/L-cell/h with negligible contribution to energy production, glutamine must be contributing primarily to anabolic processes, such as AA, nucleotide, and protein production as well as to *de novo* lipogenesis.

### Effect of Hyperglycemia/Euglycemia on Glutamine and Fatty Acid Metabolism

The MID of several TCA cycle metabolites from [U-^13^C_5_, ^15^N_2_] glutamine labeling experiments is shown in Figure 6 at different glucose concentrations in the medium – at 5 mM for euglycemia and 25 mM for hyperglycemia. With this tracer, increased glutaminolysis flux resulted in increased abundance of M + 4 mass-isotopomer of citrate, succinate, and malate (70), whereas RC resulted in increased M + 3 for malate, M + 4 for succinate, and M + 5 for citrate. RC and increased glucose contribution to the FAs production also disturbs the acetyl-CoA mass-MID. Figure 6 clearly shows that there are no significant differences in labeling patterns of all four metabolites. Consequently, based on bioreactor data, we can exclude any major change in glutaminolysis flux as well for FA production during hyperglycemia vs. euglycemia.

### Quantifying *De Novo* Lipogenesis Flux in DB-1 Cells

During culturing of DB-1 cells with [1,6-^13^C_2_] glucose, progressive labeling of FAs was observed for multiple resonances (see Figures 3 and 4D). FA synthesis occurs first by cytosolic citrate production from glucose and through RC in cytosol from glutamine through isocitrate dehydrogenase (IDH1 isoform). By ATP citrate lyase, cytosolic citrate is converted to oxaloacetate and cytosolic acetyl-CoA, which is converted further to malonyl-CoA by the rate-limiting acetyl-CoA carboxylase beta (ACC-beta) and through FAs synthases to elongated precursors via sequential addition of acetyl-CoA, eventually leading to palmitate production. Palmitate is further elongated or desaturated to produce numerous other FAs. To address *de novo* FA flux production, we utilized the BC model with cytosolic FAs and their precursors as well as membrane FAs as the second FA pool. We calculated FA concentrations based on the total FA content of approximately 20% of cell dry weight of 200 mg/10^9^ cells (5 × 10^8^ cells = 1 ml). Total FA content referenced to palmitate is at the level of several tens of mmol/L-cell. We iteratively varied the ratio of cytosolic to membrane FA and found the best fit at approximately 10 times more FA in the cytosolic membrane compartment (Figure 4D). The *de novo* flux of conversion of glucose to FA was 0.59 mmol/L-cell/h and NADPH consumption flux in FA biosynthesis was 8.4 mmol/L-cell/h.

While glutamine-derived carbons have been unlabeled, we have monitored lipogenesis by ^13^C NMR detecting methylene carbon acyl groups originating from labeled glucose (Figures 3A and 4D). Table 1 indicates that *de novo* lipogenesis corresponds to ~6% of the TCA cycle flux under hyperglycemic conditions. However, this does not include possible direct contributions from unlabeled glutamine (i.e., through cytosolic RC by isocitrate dehydrogenase IDH1 or mitochondrial IDH2) and potentially from cytosolic acetyl-CoA synthetase 2 (ACSS2) reaction, which involve participation of cytosolic acetate or acetate from the medium (78).

This modest flux for *de novo* FA synthesis requires a large contribution of NADPH cofactor production (~8.3 mmol/L-cell/h). NADPH could be produced by the oxidative branch of the PPP, ME, or IDH activities or by serine–glycine one-carbon metabolism (folate metabolism). Our calculated malic enzyme activities in mitochondria (even if all this activity is represented by ME3) would account for 4.3 mmol/L-cell/h and cytosolic NADPH flux (ME1, 0.6 mmol/L-cell/h) would not account for the required high level of NADPH flux. A recent elegant study (79) suggested that in some cancers folate metabolism is the biggest producer of NADPH. Based on these data (48), we estimated that the serine–glycine one-carbon SGOC pathway cannot account for the required NADPH consumption. In light of our ^13^C NMR lactate labeling data, the oxidative branch of the PPP is the main producer of NADPH in these cancer cells (6.6 mmol/L-cell/h). This constitutes ~80% in agreement with the NADPH consumption rate during FAs biosynthesis. Thus, the flux through the oxidative PPP appears to be ~80% sufficient to supply all of the NADPH required for FA production and is the main supplier of reducing equivalents in DB-1 melanoma. A 20% difference in the rates of NADPH production and consumption indicates that alternative sources of NADP reduction, considered above, need to be taken into account. We also found that mitochondrial-pyruvate–malate cycling was very high (4.3 mmol/L-cell/h). This could also contribute to defense against reactive oxygen species by producing sufficient NADPH via ME3.

### Lymphoma Studies

#### Rapamycin Inhibits mTORC1 Signaling and Lowers Lactate Concentration in B-Cell Lymphoma Cells

The effects of rapamycin on mTORC1 signaling and cell growth were first examined in four B-cell lymphoma cell lines, sub-classified either as germinal-center type DLBCL (DLCL2, Val, and Ly18) or Burkitt (Ramos). As indicated by loss of mTORC1-dependent S6rp phosphorylation, rapamycin suppressed mTORC1 activity in all the cell lines and profoundly inhibited BrdU uptake-detected cell proliferation (15).

**Figure 7:**
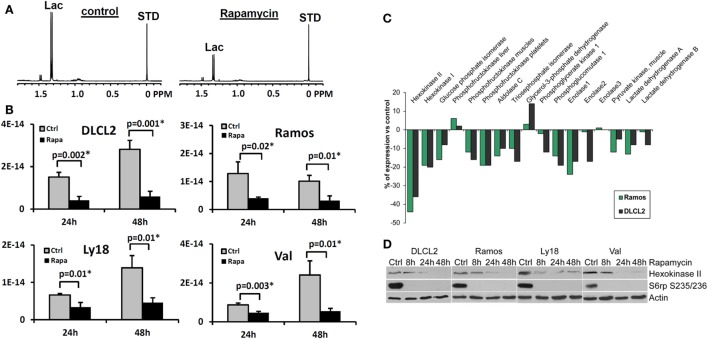
**Rapamycin-induced mTOR inhibition and changes in intracellular lactate levels and glycolytic gene expression profiles**. **(A)** 1H MRS spectra of DLCL2 cells at 24 h (control vs. rapamycin-treated); lactate (1.3 ppm) and the reference TSP peaks (0 ppm, indicated as STD). **(B)** Intracellular lactate concentration (mole/cell) for the four listed B-cell lymphoma cell lines treated with 200 nM rapamycin or medium alone for 24 and 48 h (*n* = 3 for each condition). **(C)** Expression of the glycolysis-related genes in the depicted cell lines treated for 6 h with rapamycin or medium alone. **(D)** Kinetics of the rapamycin-induced changes in hexokinase II protein expression. Inhibition of S6rp phosphorylation, an indicator of mTOR signaling, is also shown. [This research was originally published in NMR in Biomedicine. Lee et al. (15). © John Wiley & Sons Inc.]

We have evaluated the effect of rapamycin on the cellular concentration of lactate in NHL cells by ^1^H MRS (Figures 7A,B) because mTOR has been implicated in various aspects of cell metabolism. To varying extents rapamycin significantly decreased intracellular lactate relative to controls in all four cell lines (15). Significantly, the relative reduction in intracellular lactate concentrations in rapamycin treated cells vs. controls was more pronounced after 48 than 24 h exposure to rapamycin and correlated with the suppression of cell growth indicating that the effect is not directly due to drug–enzyme interaction or direct effects on mTOR signaling but is most likely due to gene translation.

#### Kinetics of the Rapamycin-Induced Suppression of Lactate Concentration

To determine how early the rapamycin-induced metabolic changes can be detected, we examined the intracellular lactate concentration at 2, 4, 8, 24, and 48 h in two B-cell lymphoma cell lines (15). While 2 and 4 h exposure to rapamycin did not have a definitive effect, we noted statistically significant differences between vehicle- and rapamycin-treated cells at 8 h in both cell lines (*p* = 0.002 and 0.03 for DLCL2 and Ramos, respectively; *n* = 3) with increased differences at 24 and 48 h. Changes of lactate and glucose concentrations in the medium indicated diminished glycolysis following rapamycin treatment with corresponding changes in lactate secretion and glucose uptake per cell. Lactate secretion and glucose uptake exhibited maximum differences between vehicle- and rapamycin-treated cell cultures at 24 h in contrast to intracellular lactate that remained approximately unchanged. The amount of secreted lactate per cell was three to four orders of magnitude greater than the intracellular lactate levels, which indicates that most of the lactate generated by cellular metabolism was secreted by the cell. Nonetheless, the level of intracellular lactate was more sensitive than secreted lactate levels for distinguishing metabolic differences between vehicle- and rapamycin-treated cells. This was, in part, due to the intracellular lactate concentration remaining essentially constant after rapamycin treatment.

#### Rapamycin Inhibits Expression of Hexokinase II

While inhibition of mTORC1 signaling by rapamycin reaches an optimum within minutes after drug administration (80–82), the drug’s effects on the lactate concentration were not detectable until 8 h post-treatment and became more pronounced at 24 and 48 h in cultured cells (15). This marked timing difference indicates that the inhibition of glycolysis does not directly result from inhibition of the mTORC1 signaling but most likely originates from diminished expression of enzymes involved in the glycolytic pathway. To test this assumption, we performed gene expression profiling of the DLCL2 and Ramos cell lines treated with rapamycin or medium alone (Figure 7C). The component of the glycolytic pathway whose gene expression was most profoundly inhibited by the rapamycin treatment was hexokinase II although some other glycolytic genes showed similar trends. The steady loss of hexokinase II expression was confirmed on the protein level in all the cell lines examined (Figure 7D).

#### Rapamycin-Induced Suppression of Lactate Concentration Directly Correlates with Inhibition of Lymphoma Growth *In vivo*

**Figure 8:**
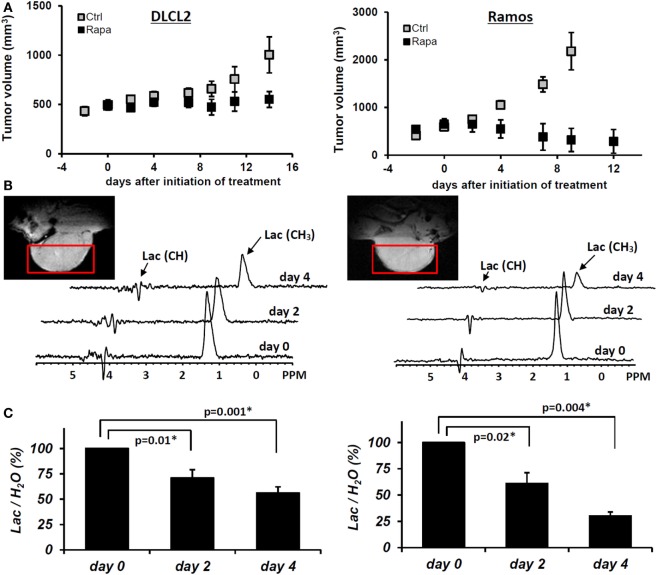
**Rapamycin-induced inhibition of tumor growth and glucose metabolism *in vivo***. Xenotransplants of DLCL2 cells (left panel) and Ramos cells (right panel). **(A)** Tumor growth curve. The data are presented as mean ± SD (*n* ≥ 5 in each group). **(B)** Time course *in vivo* localized MRS. **(C)** Lac/H_2_O ratios normalized to the pretreatment values; mean ± SE (*n* = 5). Statistically significant changes in Lac/H_2_O were observed at 48 h and after from initiation of rapamycin treatment in both xenografts while control tumors (*n* = 5) did not have significant changes in Lac/H_2_O until 96 h. [This research was originally published in NMR in Biomedicine. Lee et al. (15). © John Wiley & Sons Inc.]

To determine if the rapamycin-induced suppression of lactate concentration can also be detected *in vivo*, we evaluated xenotransplanted lymphoma models (Figure 8), the DLCL2 and Ramos B-cell lines studied in greatest detail *in vitro* as described above. The tumors displayed the appropriate transformed lymphoid cell morphology and human B-cell phenotype confirming that they originated from the implanted lymphoma cell lines.

Administration of rapamycin to mice with DLCL2 and Ramos xenografts inhibited mTORC1 signaling and decreased the proliferative rate of the lymphoma cells as determined by suppression of S6rp phosphorylation and decreased expression of the cell cycle-associated marker mib1 (15). Western blots confirmed effective and specific mTORC1 inhibition, which revealed greatly diminished S6rp phosphorylation and intact phosphorylation of Akt. Prolonged treatment with rapamycin resulted in tumor growth arrest for the DLCL2 cell line and a marked decrease in tumor volume for the Ramos cell line (Figure 8). It is noteworthy that these effects were preceded by decreases in tumor lactate concentrations measured by *in vivo* MRS. The decrease was already statistically significant at 48 h following initiation of rapamycin treatment and increased at 96 h. At 2 days after initiation of treatment, the effect of rapamycin on tumor volume was not yet noticeable while tumor lactate had already significantly decreased. These observations strongly suggest that *in vivo* measurement of lactate originating from the tumor provides an early marker predictive of effective mTOR inhibition that correlates with substantially later tumor shrinkage.

#### Discussion of Rapamycin Data

The above results clearly document that mTOR inhibition can be monitored *in vitro* and *in vivo* by measuring changes in lactate concentration. Accumulating evidence indicates that mTORC1 can be activated by a variety of cell–surface receptor complexes. While the original studies implicated the activation of receptors for the insulin-family growth factors (83–85), subsequent observations by the Wasik laboratory (80–82) and by others (86–91) demonstrated that a large spectrum of receptors and their ligands triggered mTORC1 activation. These include interleukin-2, anaplastic lymphoma kinase, CD40 ligand, notch, thyroid-stimulating hormone, fibroblast growth factor-9, polycystein-1, and prostaglandin F2a. These diverse ligand–receptor complexes activate mTORC1 through the signaling pathways PI3K/Akt (92–94), MEK/ERK (95), and possibly Syk (96). Therefore, the inhibition of these pathways should also be amenable to detection by monitoring changes in glycolysis. Given that these pathways are frequently deregulated in malignant cells and clinical-grade inhibitors are already available, evaluation of glucose metabolism as a surrogate for monitoring mTOR activity may prove useful in the management of cancer patients treated with a variety of kinase inhibitors. Indeed, inhibition of the oncogenic BCR/ABL tyrosine kinase led to decreased glucose uptake and lactate production (97, 98), all but certainly indicating that mTORC1 signaling was involved (99). Hence, the apparent lack of specificity of lactate actually reflects its versatility. Provided that a single kinase inhibitor is administered, there is no lack in specificity of detecting response to the drug; if multiple kinase inhibitors are used, then lactate still serves as a biomarker for the combination.

#### Genomic Analysis of mTOR Inhibition

In view of a recent report (100) that several metabolic pathways were modified in mouse fibroblasts with hyper-activated mTOR, we analyzed changes in expression of metabolic genes in DLCL2 and Ramos cells induced by rapamycin. Table 4 lists significantly affected (up- and downregulated; *p* < 0.02, *n* = 3) of representative metabolic genes in DLCL2 cells (Ramos data are similar). The anticipated related alterations in the cellular metabolism indicate that in the DLCL2 cells mTOR inhibition diminishes not only glucose metabolism but also TCA cycle activity, *de novo* FA and sterol biosynthesis, pyruvate cycling activity, and flux through pentose phosphate pathway. Glutaminolysis should also be affected due to novel PPAT gene expression, which is responsible for enzymatic glutamate production from glutamine (see footnote “b” in Table 4).

**Table 4:** **Changes in gene expression following treatment of DLCL2 cells with rapamycin**.^a^

Entrez ID	Gene Symbol	Enzyme name	EC number	Fold change	*P value*	Pathway Affected
**Upregulated**
5106	*PCK2*	Phosphoenolpyruvate carboxykinase 2 (mit)	EC 4.1.1.32	1.51	0.000434	Pyruvate cycling
**Downregulated**
3099	*HK2*	Hexokinase 2	EC 2.7.1.1	−1.78	0.00515	Glycolysis
57546	*PDP2*	Pyruvate DH phosphatase subunit 2	EC3.1.3.43	−1.54	0.0150	PDH, TCA
3419	*IDH3A*	Isocitrate DH 3 (NAD +) alpha (mito)	EC1.1.1.41	−1.60	0.000673	TCA Cycle
5471	*PPAT*	Amidophosphoribosyl transferase	EC2.4.2.14	−1.73	0.0163	Glutaminolysis^b^
31	*ACACA*	Acetyl-CoA carboxylase alpha	EC6.3.4.14	−1.80	0.00518	Fatty acids
2180	*ACSL1*	Acyl-CoA synthetase long-chain member 1	EC 6.2.1.3	−1.52	0.0161	Fatty acids
23171	*GPD1L*	Glycerol-3-phosphate dehydrogenase 1-like	EC 1.1.1.8	−1.56	0.00446	Glycerophospholipid
22934	*RPIA*	Ribose 5-phosphate isomerase A	EC 5.3.1.6	−1.58	0.00451	Pentose phosphate
1717	*DHCR7*	7-dehydrocholesterol reductase	EC1.3.1.21	−1.72	0.00363	Cholesterol
6307	*MSMO1*	Methylsterol monooxygenase 1	EC1.14.13.72	−1.74	0.0165	Cholesterol
1723	*DHODH*	Dihydroorotate dehydrogenase	EC 1.3.5.2	−1.51	0.000190	Pyrimidine metabolism
5198	*PFAS*	Phosphoribosylformylglycinamidine synthase	EC 6.3.5.3	−1.51	0.00120	Purine metabolism

*^a^Data were compiled from KEGG database. Gene expression of a large number of enzymes demonstrated statistically significant changes, but only one or two representative enzymes are listed for pathways showing changes greater than 50%*.

*^b^This is a new link for glutamine to glutamate interconversion through operation of enzyme 5-phospho-β-d-ribosylamine:diphosphate phospho-α-d-ribosyl-transferase (glutamate-amidation reaction)*.

#### Cell Perfusion and ^13^C NMR Spectroscopy

**Figure 9:**
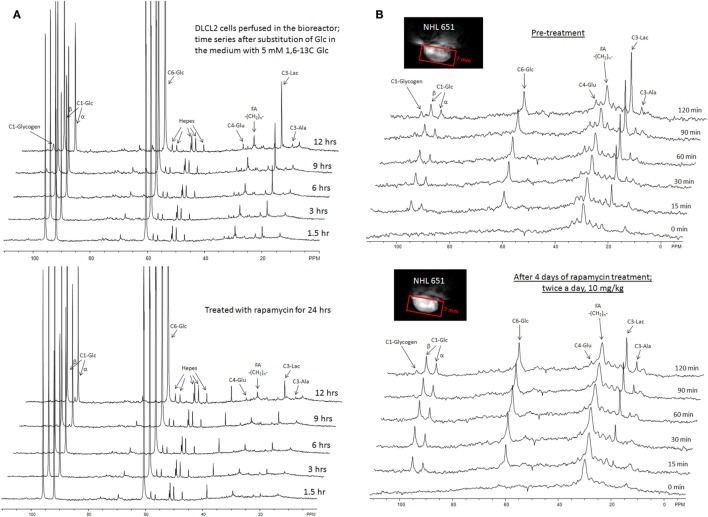
**Time course ^13^C NMR spectra of perfused DLCL2 lymphoma cells and *in vivo* tumors upon rapamycin treatment**. **(A)** Perfused DLCL2 cells in 9.4T/8.9 cm vertical bore Varian NMR spectrometer. 4 × 10^8^ cells were encapsulated in agarose and perfused in a 10 mm diameter NMR perfusion chamber with RPMI 1640 medium containing 5 mM [1,6-^13^C_2_] glucose maintained under an atmosphere of 20% O_2_, 75% N_2_, 5% CO_2_ at 37°C. **(B)** *In vivo* ^13^C MRS spectra of a nude mouse with 12 mm DLCL2 tumor implanted in its flank. Spectra were measured before and after rapamycin treatment with seven consecutive doses (10 mg/kg) administered by oral gavage twice daily. During the study, 450 mM [1,6-^13^C_2_] glucose was infused through the tail vein at variable infusion rates. Each spectrum was acquired in 15 min on a 9.4 T/31 cm spectrometer with a home-built 13 mm loop-gap resonator.

Figure 9A shows data obtained from DLCL2 cells immobilized in agarose beads and perfused with normoxic (20% O_2_, 75% N_2_, 5% CO_2_) media containing 5 mM [1,6-^13^C_2_] glucose over a 12-h period in a 10 mm diameter NMR bioreactor. Cells were studied before treatment and 24 and 48 h (not shown) after treatment with rapamycin. Using a two-compartmental enzyme kinetic model, we calculated lactate production rates. The results were 6.9 mM/h (pretreatment) and 1.6 mM/h (post-treatment), which indicate a profound decrease in glycolysis. There is also a decrease in labeling of Glu C4 reflecting a decrease in TCA cycle flux. Alanine and fatty acid labeling are also diminished, and glycogen stores have been completely depleted, probably as a result of decreased energy production by both glycolytic and TCA cycle pathways.

#### *In vivo* ^13^C NMR Studies of DLCL2 Cell Metabolism

Figure 9B shows the time course of ^13^C MR spectra measured after infusion of [1,6-^13^C_2_] glucose into a mouse with a 12 mm DLCL2 xenograft (500 mm^3^) growing on its flank. The inset to this figure shows the MR image of the tumor and the Hadamard voxel that localizes the spectral data in the tumor (i.e., insuring no contributions from external tissues). These studies demonstrate the feasibility of performing ^13^C MRS studies non-invasively in these *in vivo* lymphoma tumor models in mice. The spectra show the time course of labeling of tumor metabolites by transfer of ^13^C by metabolism of the labeled glucose over 120 min with each spectrum measured in 15 min. The same animal was studied before and 4 days later after seven doses of rapamycin (10 mg/kg) with two doses administered per day (bottom of Figure 9B). The rate of lactate labeling has decreased, whereas the rate of alanine labeling appears to have increased. Glutamate-C4 was detectable and appears to have decreased after rapamycin treatment, suggesting a decrease in TCA cycle flux. The glycogen peak appears very small in both sets of spectra and seems to have been diminished but not eliminated after treatment. These are among the first *in vivo* spectra we or anyone else we are aware of have reported. We are pursuing a number of strategies for improving the resolution and S/N ratio. Obviously, higher magnetic fields or cryoprobes would increase the sensitivity, but even with the equipment at hand it is clearly feasible to obtain quantitative data *in vivo*.

**Figure 10:**
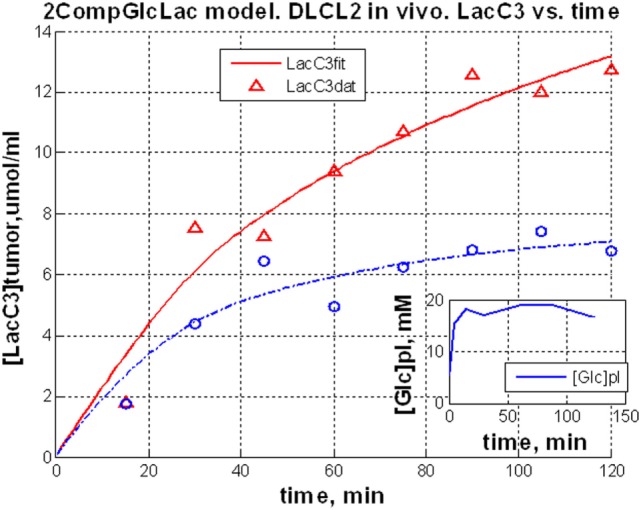
**Dynamic glycolysis model of subcutaneous DLCL2 in a mouse**. Fitting of LacC3 *in vivo* data vs. time. Triangle, pretreatment, and circle, posttreatment. Total lactate production rate, Flac = 0.80 mM/min (pretreatment) and Flac = 0.62 mM/min (posttreatment). Clearance rates were Fcl = 0.59 mM/min (pretreatment) and Fcl = 0.56 mM/min post-treatment (non-steady-state condition). Rapamycin treatment affects mostly the lactate production rate. The inset is a typical blood glucose input function used in the model.

A computational analysis (Figure 10) of the time course *in vivo* data from a mouse to a two-compartment non-steady-state enzyme kinetic model generated total lactate production and clearance rates (indicated in the figure caption). The model included glycolysis, alanine production, and exchange of cytosolic and vascular/interstitial lactate through MCT1 (the dominant monocarboxylic acid transporter). The calculated fluxes indicate a decrease in glycolytic metabolism caused by treatment with rapamycin. Clearly a more comprehensive analysis, including other metabolic pathways that contribute to lactate production is required, but this demonstrates feasibility of our precise data analysis approach.

#### LC-MS Studies of DLCL2 Tumor Cells

**Figure 11:**
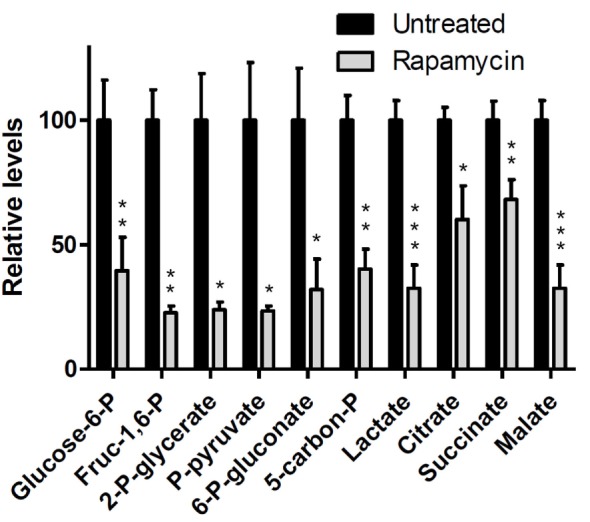
**Mass spectrometric data of intracellular metabolites**. DLCL2 tumor cells were treated with and without 200 nM rapamycin for 48 h. Measurements were made at steady-state isotope exchange conditions (*n* = 3 for each condition). *p < 0.05, **p < 0.01, ***p < 0.001.

Preliminary studies of DLCL2 cells in suspension culture have been initiated in Dr. Blair’s laboratory (Figure 11). Cells were grown in batch culture. Harvested cells were spiked with [U-^13^C] lactate, citrate, and succinate as internal standards. The extracted metabolites were separated by a reverse-phase ion-paring chromatography and analyzed by MS. Note that these are steady-state measurements, which only provide information about relative fluxes. Kinetic data are required for absolute flux measurements. Significant changes are evident in levels of glycolytic metabolites (glucose-6P, fructose-1,6-P, 2-phosphoglycerate, pyruvate, and lactate), pentose shunt metabolites (6-phosphogluconate and 5-carbon phosphates), and TCA cycle (citrate, succinate, and malate) (100, 101).

In view of the recent interest in glutamine metabolism of tumors (71), preliminary studies were performed on DLCL2 cells grown in culture with 4 mM [U-^13^C] glutamine; after 6 h steady state was achieved. A preliminary analysis fitted the LC-MS isotopomer data (aspartate, glutamate, lactate, malate, succinate, citrate, and α-ketoglutarate, Mo, M_1, …,_ M*_n_*, *n* = number of carbons) to all the metabolic pathways included in the fragmented cumomer model (Shestov et al., manuscript in preparation) (13, 48, 49). There was excellent agreement between observed and calculated isotopomer levels (data not shown). The metabolic network includes all the pathways included in the bonded cumomer model (Figure 1). Table 5 summarizes changes in flux through various pathways for which there was adequate significance. Rapamycin produced dramatic changes in RC (41% decrease), glutaminolysis (95% decrease), MPC (50% increase) and PC (520% increase). Clearly, further study of these effects is required, but these data strongly suggest that multiple pathways are affected and, potentially, may serve as key biomarkers of effective mTOR inhibition.

**Table 5:** **Pathway activities for DLCL2 cells based on ^13^C LC-MS data**.^a^

Pathway name	Control	Rapamycin	*P-value*
Reductive carboxylation (cytosolic)	0.29 ± 0.05	0.17 ± 0.03	0.0235
Glutaminolysis	0.27 ± 0.10	0.014 ± 0.02	0.0122
Mitochondrial pyruvate carrier	0.63 ± 0.06	0.90 ± 0.09	0.0124
Pyruvate dehydrogenase complex	0.60 ± 0.07	0.56 ± 0.05	0.466
Pyruvate carboxylase (mitochondrial)	0.075 ± 0.030	0.39 ± 0.09	0.0045
*De Novo* fatty acid production	0.39 ± 0.07	0.23 ± 0.05	0.0322

*^a^Presented as real parameter value ± error in terms of standard deviation (SD). Errors are based on Monte Carlo simulations assuming Gaussian noise with 0 mean and σ = 0.01 for SD; synthetic renormalized steady-state mass-isotopomers fit by fragmented cumomer model using Simplex*.

## Conclusion and Summary

We have for the first time formulated and validated a detailed metabolic network model of tumor intermediary metabolism that includes all the key pathways except glycogen and phospholipid metabolism as well as serine–glycine one-carbon metabolism. These pathways can be added in the future. We have demonstrated that glutamine metabolism, while critical to AA and nucleoside/tide production, makes minimal contribution to energy production in DB-1 melanoma. Studies of FA metabolism have been initiated and need to be advanced further. Aerobic glycolysis and oxidative metabolism contribute approximately equally to energy production in this tumor and probably contribute in similar proportions to energy production by many tumors, with relative contributions of glycolysis being modulated by oxygen supply in the local environment of the tumor.

Using NHL as a model and rapamycin as a specific therapeutic agent, we have formulated a general strategy for monitoring therapeutic response of signal transduction. This strategy includes preliminary exploratory non-invasive ^13^C MRS studies of tumor *in situ* and *ex vivo* ^13^C MRS and LC-MS studies of tumor metabolism to identify key pathways that are modified by treatment of individual patients with specific inhibitors. Because of the intrinsic low sensitivity of ^13^C MRS, a key goal of these exploratory studies will be to identify biomarkers of therapeutic response that can be more readily studied by more sensitive methods, such as ^1^H MRS and CEST (102) and PET/CT. In this regard, lactate appears to be a critical metabolite that plays a central role in a number of key metabolic pathways susceptible to modulation by signaling inhibitors. Dynamic nuclear polarization may also play an important role, although the short lifetime of probes, such as ^13^C-labeled pyruvate, forces them to be employed at abnormally high concentrations, which may modify the metabolic pathways that are being monitored. Despite its intrinsically low sensitivity, ^13^C MRS of glucose, lipids as well as low molecular weight metabolites, such as acetate and glutamine, should not be ignored, especially in view of the increasing availability of high-field instruments, such as 7 Ts, of which more than 60 are already available at leading institutions throughout the world. These are likely to soon gain FDA approval and will become much more useful as body coils and other probes become more available. The recent demonstration of the feasibility of *ex vivo* ^13^C MRS and LC-MS analysis of tumor specimens that have been surgically excised (18, 19) in conjunction with the current *in vivo* ^13^C MRS study at 9.4 T of human melanoma and lymphoma xenografts in mice point to the feasibility of non-invasive human metabolic studies. Overall, metabolic studies are likely to complement genomic studies of human cancer in the near future.

## Author Contributions

The study was conceived and coordinated by JG and AS; SL, KN, LG, DN, and JR designed and performed the experiments, and collected the data; AS, SL, KN, LG, IB, and JG analyzed and interpreted the experiments; AS performed computational metabolic analysis; MW and DL provided technical assistance and contributed to the preparation of the manuscript; JG provided conceptual advice; JG, SL, and AS wrote and edited the manuscript with the assistance of all authors.

## Conflict of Interest Statement

The authors declare that the research was conducted in the absence of any commercial or financial relationships that could be construed as a potential conflict of interest. The reviewer NM and handling Editor declared their shared affiliation and the handling Editor states that the process nevertheless met the standards of a fair and objective review.

## Abbreviations

AA, amino acids; ASCT2, alanine-serine-cysteine (GLN) transporter 2; BC, Bonded Cumomer; CEST, chemical exchange saturation-transfer; CMR, cellular metabolic rate; CMRatp, ATP production rate; CMRlac, Lactate production rate; FA, fatty acid; GLUT, glucose transporter; h, hours; IDH, isocitrate dehydrogenase; MCT, monocarboxylic acid transporter; MID, mass-isotopomer distribution; MRO_2_, O_2_ consumption rate; oxphos, oxidative phosphorylation; PPP, pentose phosphate pathway; P/O, phosphate to oxygen ratio; RC, reductive carboxylation; SGOC, serine glycine one carbon metabolism; TCA cycle, tricarboxylic acid cycle.
